# ENU-induced Mutation in the DNA-binding Domain of KLF3 Reveals Important Roles for KLF3 in Cardiovascular Development and Function in Mice

**DOI:** 10.1371/journal.pgen.1003612

**Published:** 2013-07-11

**Authors:** Lois Kelsey, Ann M. Flenniken, Dawei Qu, Alister P. W. Funnell, Richard Pearson, Yu-Qing Zhou, Irina Voronina, Zorana Berberovic, Geoffrey Wood, Susan Newbigging, Edward S. Weiss, Michael Wong, Ivan Quach, S. Y. Sandy Yeh, Ashish R. Deshwar, Ian C. Scott, Colin McKerlie, Mark Henkelman, Peter Backx, Jeremy Simpson, Lucy Osborne, Janet Rossant, Merlin Crossley, Benoit Bruneau, S. Lee Adamson

**Affiliations:** 1 Samuel Lunenfeld Research Institute of Mount Sinai Hospital, Toronto, Ontario, Canada; 2 Centre for Modeling Human Disease, Toronto Centre for Phenogenomics, Toronto, Ontario, Canada; 3 School of Biotechnology and Biomolecular Sciences, University of New South Wales, Sydney, New South Wales, Australia; 4 Mouse Imaging Centre, Toronto Centre for Phenogenomics, Toronto, Ontario, Canada; 5 The Hospital for Sick Children Research Institute, Toronto, Ontario, Canada; 6 Department of Medical Biophysics, University of Toronto, Toronto, Ontario, Canada; 7 Department of Molecular Genetics, University of Toronto, Toronto, Ontario, Canada; 8 Heart and Stroke Richard Lewar Centre of Excellence, Toronto, Ontario, Canada; 9 Department of Physiology, University of Toronto, Toronto, Ontario, Canada; 10 Department of Medicine, University of Toronto, Toronto, Ontario, Canada; 11 Gladstone Institute of Cardiovascular Disease, Department of Pediatrics, and Cardiovascular Research Institute, University of California, San Francisco, California, United States of America; 12 Department of Obstetrics and Gynaecology, University of Toronto, Toronto, Ontario, Canada; Seattle Children's Research Institute, United States of America

## Abstract

KLF3 is a Krüppel family zinc finger transcription factor with widespread tissue expression and no previously known role in heart development. In a screen for dominant mutations affecting cardiovascular function in N-ethyl-N-nitrosourea (ENU) mutagenized mice, we identified a missense mutation in the *Klf3* gene that caused aortic valvular stenosis and partially penetrant perinatal lethality in heterozygotes. All homozygotes died as embryos. In the first of three zinc fingers, a point mutation changed a highly conserved histidine at amino acid 275 to arginine (*Klf3^H275R^*). This change impaired binding of the mutant protein to KLF3's canonical DNA binding sequence. Heterozygous *Klf3^H275R^* mutants that died as neonates had marked biventricular cardiac hypertrophy with diminished cardiac chambers. Adult survivors exhibited hypotension, cardiac hypertrophy with enlarged cardiac chambers, and aortic valvular stenosis. A dominant negative effect on protein function was inferred by the similarity in phenotype between heterozygous *Klf3^H275R^* mutants and homozygous *Klf3* null mice. However, the existence of divergent traits suggested the involvement of additional interactions. We conclude that KLF3 plays diverse and important roles in cardiovascular development and function in mice, and that amino acid 275 is critical for normal KLF3 protein function. Future exploration of the KLF3 pathway provides a new avenue for investigating causative factors contributing to cardiovascular disorders in humans.

## Author Summary

Cardiac defects are among the most common malformations in humans. Most causative genetic mutations remain unknown. To discover new causative genes important in cardiovascular development and function, we examined 1770 mice with randomly mutated genes and found a mutant with aortic valvular stenosis, and increased risk of fetal and neonatal death. Using linkage analysis and sequencing, we identified a protein-altering point mutation in the gene regulatory protein KLF3. Mice that survived into adulthood with one mutant copy of the *Klf3* gene had low arterial blood pressure, enlarged hearts, and increased mortality due to heart failure. When both copies of the *Klf3* gene was mutant, then embryos had heart defects, and all died before birth. KLF3 had no previously known role in heart development so to confirm these findings, we (1) knocked down *klf3* expression in zebrafish embryos and (2) examined mice with a mutation that effectively eliminated the KLF3 protein. In both cases, cardiovascular dysfunction was observed. In conclusion, we have discovered that KLF3 plays diverse and important roles in cardiovascular development and function in mice. Future exploration of the KLF3 pathway provides a new avenue for investigating causative factors contributing to cardiovascular disorders in humans.

## Introduction

Congenital heart defects are the most common congenital malformations in humans affecting 1–2% of live births [1] and 18% of stillbirths [2]. Causative mutations have been identified in families with inherited congenital heart defects [3] but in most cases remain unknown [2]. A strong genetic role is nevertheless likely given high heritability scores, for example >0.7 for left-sided congenital heart defects [4], [5], [6]. To discover new genes important in cardiovascular development, we measured aortic blood velocity in an ultrasound screen undertaken to assess left ventricular outflow function, in the offspring of N-ethyl-N-nitrosourea (ENU) mutagenized male mice [7]. One mutant had very high aortic blood velocities due to aortic valvular stenosis and this trait was heritable. Additional abnormalities in cardiovascular development and function were found in subsequent phenotyping of this mutant mouse line. A dominant point mutation in the region encoding the DNA binding domain of *Klf3* was found by linkage analysis and gene sequencing. KLF3 is a zinc finger transcription factor that has discrete regions of expression that are widely distributed among embryonic and adult tissues in mice [8], [9]. KLF3 functions predominantly as a gene repressor [10] although it also has activator functions [11].

KLF3 had hitherto no described role in heart or vascular development or function. In prior work, homozygous deletion of the region encoding the *Klf3* zinc finger DNA binding domain caused partially penetrant perinatal lethality in mice and significant abnormalities in adiposity [12], B cell development [13], and erythroid maturation [14] whereas cardiovascular defects were not reported. However, embryonic lethality [12] occurred at a stage of development consistent with death due to cardiovascular dysfunction [15]. Furthermore, several *Klf*s are expressed in cardiomyocytes and vascular smooth muscle cells [16] including *Klf3* (current study and [17]). KLF3 is enriched at promoters of several muscle-specific genes including *muscle creatine kinase (MCK)* where it interacts with Serum Response Factor to act as a transcriptional activator [11]. Thus, despite known molecular mechanisms whereby KLF3 may alter cardiac or vascular development or function at a cellular level, a cardiovascular phenotype remained unidentified.

Herein we report the characterization of a new ENU-induced mouse mutant. Results reveal important and novel roles for KLF3 in cardiovascular development and function. Strong similarities in phenotype with homozygous *Klf3* gene trap mice, where KLF3 is largely eliminated, suggest a predominantly dominant negative effect of the point mutant protein. However, intriguingly, the existence of divergent traits suggests the involvement of additional interactions. At the molecular level, the point mutation illuminates the critical importance of a highly conserved residue in the DNA binding domain of KLF3. The discoveries reported here provide impetus for exploring the KLF3 pathway to discover new causative factors contributing to cardiovascular disorders in humans.

## Results

### Breeding and mapping of the mutation

**Figure 1:**
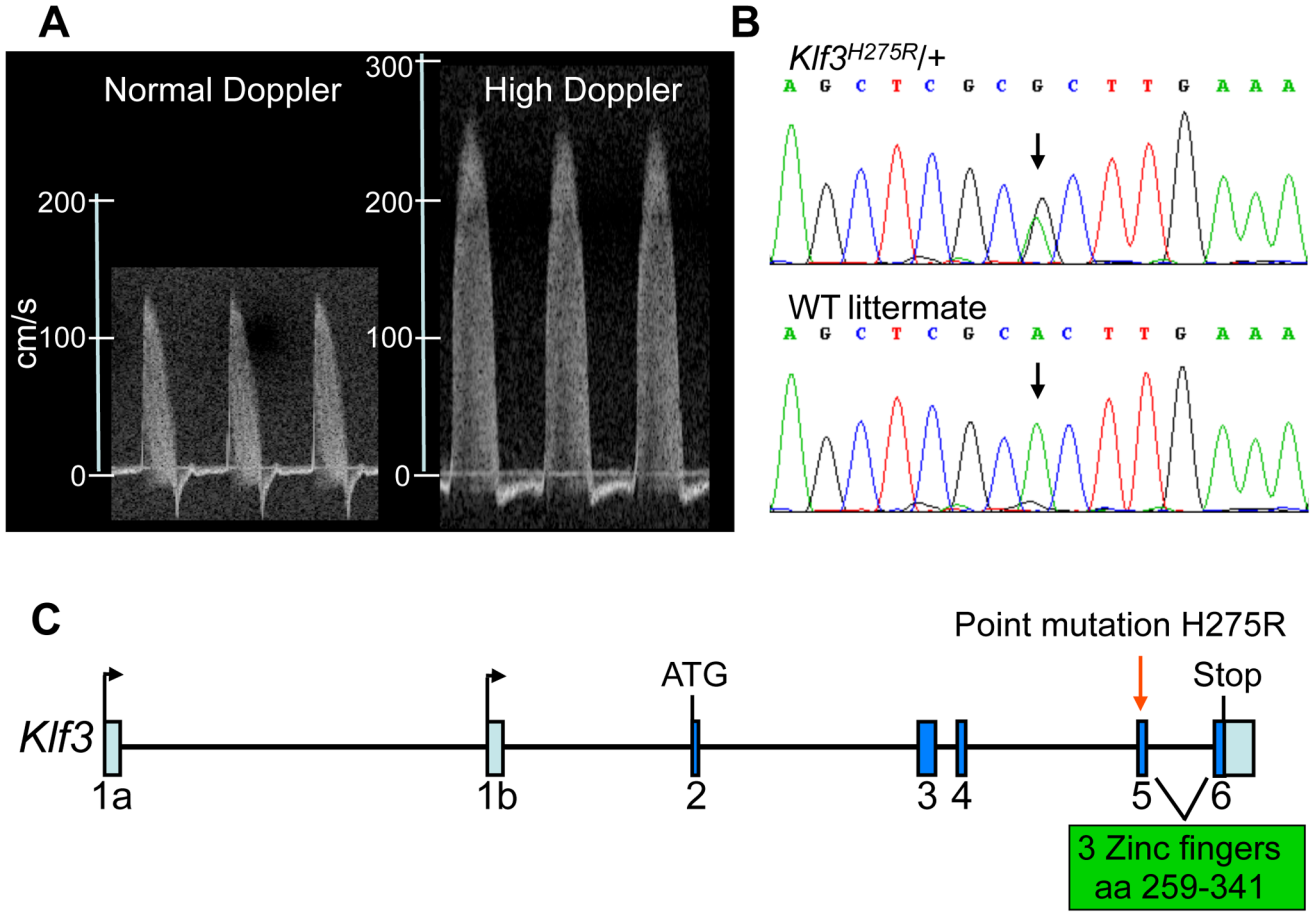
Mapping and identification of *Klf3* ^H275R^ mutation. (A) Example of the ascending aortic blood velocity waveforms in an adult mouse with a normal waveform and one with the high Doppler trait that was used to identify mutants for mapping. (B) Sequencing of the candidate *Klf3* gene revealed an A to G transition (at black arrows) in mice with mutant trait (top) but not in WT littermates (below) or in parental strains (not shown). This transition converts histidine to arginine at amino acid 275 in the KLF3 protein. (C) The location of the H275R point mutation (red arrow) is in exon 5 in the zinc finger region (amino acids 259–341) of the *Klf3* gene.

In a screen of 1770 adult heterozygous offspring from ENU mutagenized C57BL/6J male mice crossed with wild-type (WT) C3H/HeJ females, we identified a mutant mouse with an aortic blood velocity >7 standard deviations (SD) above the mean. Using a cut-off aortic blood velocity of 150 cm/s (i.e. >3 SD above the mean of all animals), we found that the trait (**Figure 1A**) was heritable when mutants were bred to BALB/cJ females although only 10% (17 of 165) had the trait. Nevertheless linkage analysis localized the mutation to chromosome 5 between 4.9 and 75.6 Mb (**Figure S1A**). The LOD score exceeded 4 in this interval (**Figure S1B)** whereas it was <2.5 elsewhere in the genome (not shown). The incidence of the trait was higher on a C57BL/6J (B6) background (136 of 584; 23%) so we performed fine mapping by crossing affected animals with B6–Chr 5 A/J consomic mice (incidence of trait was 40 of 183; 22%). We narrowed the interval to a 12.6 Mb region on chromosome 5 (**Figure S1B**), which contained 35 genes (**Table S1**). Genomic sequencing of 7 candidates (**Table S1**) revealed only one point mutation predicted to affect the protein product (**Figure 1B**). The mutation in exon 5 of *Klf3* (*Krüppel-like factor 3*) (**Figure 1C**) changed a histidine residue (CAC) at amino acid 275 to arginine (CGC) (*KLF3^H275R^*) (**Figure 1B**). This histidine is conserved across species (www.ncbi.nlm.nih.gov/homologene) and across all but one of the 22 *Sp/Klf* family members [18]. It is the central of 3 amino acids predicted to make contact with DNA in the DNA binding region of the first of three zinc finger domains in KLF3 [18], [19]. We predicted that mutation at this site would be highly likely to affect the DNA binding function of KLF3 and thereby its function in transcriptional control. The *Klf3* ^H275R^ line was subsequently maintained by breeding with B6 mice.

### Aortic valvular stenosis in adult heterozygous Klf3 point mutants

**Figure 2:**
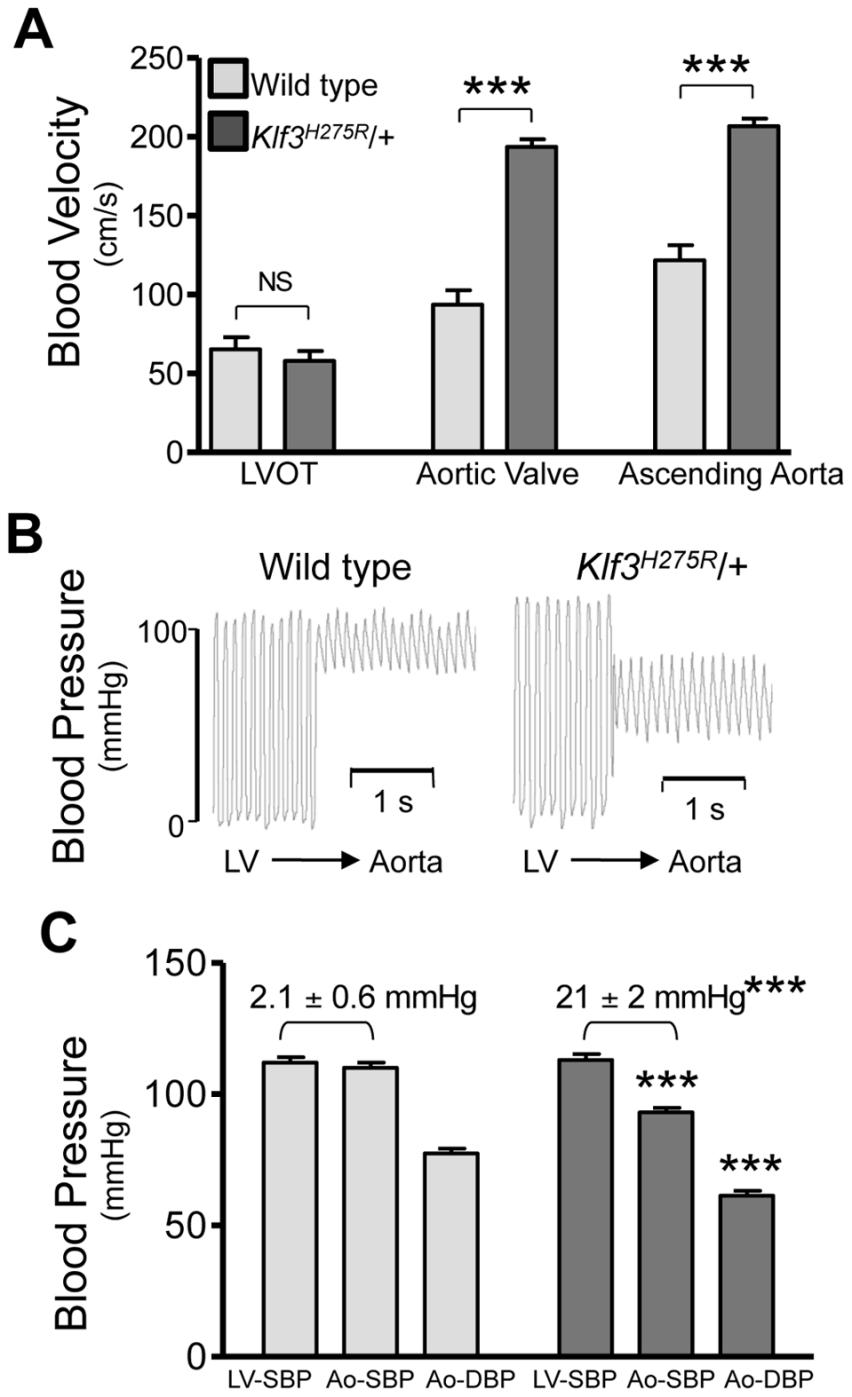
Hemodynamic evidence for aortic valvular stenosis in *Klf3^H275R^*/+ mice. Adult *Klf3^H275R^*/+ mice with high aortic blood velocities and their cage-mate controls were studied. (**A**) Doppler ultrasound was used to measure transvalvular blood velocity in *Klf3^H275R^/*+ mutants (dark bars) relative to WT (light bars). Blood velocity in the left ventricular outflow tract (LVOT) was similar in mutants and WT but became elevated at the level of the aortic valve and remained elevated in the proximal ascending aorta (24–33 wk adult mice, n = 5 per group, mean ± SE). (**B**) The transvalvular pressure gradient was measured by pulling a catheter from the left ventricle (LV) to the aorta. The gradient in systolic blood pressure from the LV to the aorta was greater in *Klf3^H275R^*/+ mutant (right) than WT mice (left). (**C**) Left ventricular systolic blood pressures (LV-SBP) were similar but aortic systolic (Ao-SBP) and aortic diastolic (Ao-DBP) blood pressures were lower in *Klf3^H275R^*/+ mutants (dark bars on right) than WT mice (light bars on left). The LV-SBP to Ao-SBP gradient (mean ± SE above bars) was significantly greater in mutants suggesting aortic stenosis (18–25 wk adult mice, n = 10 WT, n = 9 mutants, mean ± SE). *** P<0.001 *Klf3^H275R^*/+ vs. WT.

**Figure 3:**
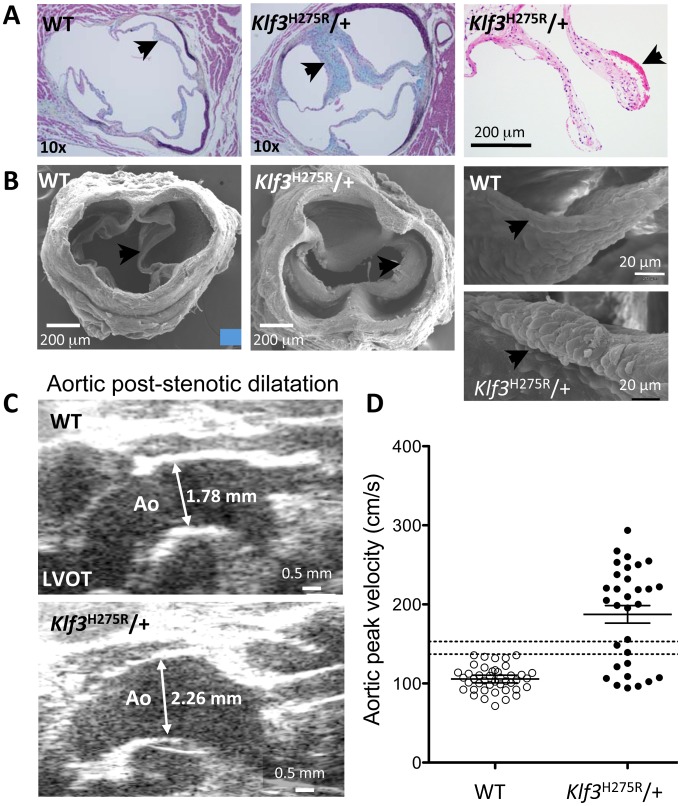
Morphological evidence for aortic valvular stenosis in *Klf3* ^H275R^/+ mice. (A) Histological sections showing thickened aortic valve leaflets in adult *Klf3* ^H275R^/+ (arrow in center and right panels) vs. WT (arrow in left panel). Blebs or hematomas were sometimes observed on aortic valve leaflets of *Klf3* ^H275R^/+ mice (e.g. arrow in right panel). (B) Scanning electron microscopy images of adult aortic valves (arrows) from WT (left) and *Klf3* ^H275R^/+ (center), with magnified images of valve leaflets (right), that show thickened leaflets in *Klf3* ^H275R^/+ mice. (C) Micro-ultrasound images of aortic arch (Ao) in adult *Klf3* ^H275R^/+ (below) vs. WT (above) showing aortic dilatation in the left ventricular outflow tract (LVOT) typical of *Klf3* ^H275R^/+ mice. (D) Elevated peak blood velocity in the ascending aorta of *Klf3* ^H275R^/+ (solid circles) relative to WT controls (open circles). Mean ± SE are shown for each group. The lower dashed line shows the WT mean + 2SD and the upper dashed line the WT mean + 3SD. 20 of 31 *Klf3* ^H275R^/+ had peak velocities >3SD above WT.

High peak aortic blood velocity in adult heterozygous *Klf3* point mutants (*Klf3^H275R^*/+) was caused by aortic valvular stenosis as shown by augmented valvular gradients in blood velocity (**Figure 2A**) and blood pressure in *Klf3* ^H275R^/+ mice (**Figure 2B,C**), and by abnormal valve morphology detected by gross dissection (not shown), histopathology (**Figure 3A**), and scanning electron microscopy (**Figure 3B**). Aortic valves were tricuspid although bicuspid valves were occasionally observed. The leaflets were thickened, often partially fused, and sometimes exhibited blebs or small hematomas (**Figure 3A**). When genotype was used to identify mutants, most *Klf3* ^H275R^/+ mice had peak velocities >150 cm/s (20 of 31 or 65%) in contrast with WT littermates (0 of 43 or 0%) (**Figure 3D**). Males and females were similarly affected. Significant aortic valve regurgitation was not observed. In humans, aortic valvular stenosis is often associated with post-stenotic aortic dilatation [20]. We therefore measured diastolic diameter of the ascending aorta *in vivo* and found a significant 27% post-stenotic enlargement in male and female *Klf3* ^H275R^/+ mice (1.95±0.07 vs. 1.53±0.08 mm in males (n = 5) and 1.70±0.09 vs. 1.34±0.03 mm in females (n = 4); P<0.01) (**Figure 3C**).

We next examined blood velocities through the other heart valves. Peak blood velocity was ∼30% higher in the main pulmonary artery, and at the atrioventricular valves during early ventricular filling (E-wave) in *Klf3* ^H275R^/+ mice (**Table 1**). E-wave fusion with the atrial filling wave (A-wave) occurred significantly more often in the left or right filling waveforms in *Klf3* ^H275R^/+ (9 of 20) than WT littermates (0 of 20). There were no abnormalities in peak inflow velocities during atrial contraction (i.e. A-wave) or in heart rate (**Table 1**), and no evidence of significant valve regurgitation in Doppler waveforms (not shown). No structural abnormalities in the pulmonary valves (**Figure S2A,B**) or the atrioventricular valves (not shown) were detected in adults by gross or histopathology examination. Thus, the mutation appeared to predominantly impact the aortic semilunar valve.

**Table 1:** Adult cardiac parameters measured by micro-ultrasound echocardiography.

		*Klf3* ^H275R^ point mutant		XS *Klf3* gene trap mutant			CH *Klf3* gene trap mutant		
		WT (10)	Het (10)	WT (9)	Het (5)	Homo (7)	WT (6)	Het (6)	Homo (8)
**BW**	g	30±2	26±1**	35±5	35±5	26.9±0.9	34±4	34±5	29±2
**HR**	min^−1^	462±8	487±11	479±20	435±19	455±13	454±21	452±18	459±17
**PV-Ao**	cm s^−1^	101±3(5)	262±24*** (5)	116±5	131±16	179±33	98±6	110±2	206±17*
**CO**	ml min^−1^	12.6±0.9	22±1***	11.8±0.5	12±2	17±3*	11.0±0.9	10.4±0.9	22±1***
**LV-ESV**	µl	33±3	38±4**	24±4	28±4	27±6	22±3	25±8	28±3
**LV-EDV**	µl	60±4	83±6***	50±5	54±6	65±12	46±4	48±7	76±5*
**EF**	%	45±3	55±2**	53±4	49±6	60±3	54±4	52±7	64±3
**LVWAd**	mm	0.90±0.04	0.93±0.04	0.98±0.07	1.0±0.1	0.88±0.06	1.04±0.07	1.00±0.07	1.01±0.06
**LVWPd**	mm	0.75±0.03	0.83±0.04	0.88±0.05	0.91±0.07	0.91±0.07	0.79±0.02	0.78±0.05	0.95±0.03
**LV E-wave**	cm s^−1^	75±1	95±1*** (5#)	69±3	69±3	68±6(6)	67±2(5)	58±5(4)	78±6(7)
**LV A-wave**	cm s^−1^	54±2	53±9 (5#)	51±3	46±3	51±4(6)	43±3(5)	44±5(4)	60±6(7)
**LV E/A**		1.41±0.07	2.0±0.4 (5#)	1.39±0.05	1.53±0.09	1.33±0.02 (6)	1.6±0.1 (5)	1.4±0.1 (4)	1.3±0.1 (7)
**ICT**	ms	9.2±0.6	7.1±0.3*(9)	8.8±0.6	10±1	9±1(6)	10±2	10±1	10±1
**IRT**	ms	13.3±0.6	10.0±0.4***(9)	11.1±0.4	13±1	12.6±0.9 (6)	13.3±0.7	15±4	13±1
**ET**	ms	48±2	53±1* (9)	50±2	51±1	51±2(6)	55±3	53.7±0.9	51±2
**LV Tei Index**		0.48±0.03	0.32±0.01***	0.40±0.01	0.45±0.04	0.44±0.04	0.43±0.03	0.46±0.06	0.47±0.03
**LA Area**	mm^2^	5.4±0.4 (5)	8.0±0.4**(5)	4.3±0.4	4.4±0.4	5.4±0.8	4.4±0.2	4.3±0.6	7.6±0.8*** (7)
**PV-PA**	cm s^−1^	70±2(5)	91±3*** (5)	76±4	82±9	95±7	74±6	79±3	110±14*
**RV E-wave**	cm s^−1^	27±2	36±2*** (6#)	26±1	29±1 (4)	31±2(6)	27±3(5)	26±2 (5)	40±5**(6)
**RV A-wave**	cm s^−1^	44±1	50±4 (6#)	47±2	52±2 (4)	51±3(6)	44±4(5)	44±2 (5)	54±3(6)
**RV E/A**		0.61±0.03	0.72±0.03* (6#)	0.55±0.01	0.57±0.02(4)	0.61±0.02*(6)	0.62±0.04 (5)	0.59±0.08(5)	0.74±0.08 (6)
**RA Area**	mm^2^	7.9±0.4 (5)	11.1±0.6**(5)	8.0±0.5	9.1±0.8	13±2*	7.7±0.5	9±1	18±1***
**Age**	wk	19±1	19±1	21±4	16±3	22±6	33±9	33±10	34±2

Mean ± SE, N is shown in parentheses in column heading or in cell if different, missing N was due to fusion of E and A waves (indicated by #) or for other technical reasons.

*** P<0.001,

** P<0.01,

* P<0.05 vs. WT; results were compared with age-matched WT mice with same background (usually littermates).

WT, wild type; Het, heterozygous; Homo, homozygous.

BW, body weight; HR, heart rate; PV-Ao, peak velocity in ascending aorta; CO, cardiac output; LV, left ventricle; ESV, end-systolic volume; EDV, end-diastolic volume; EF, ejection fraction; LVWAd, left ventricular wall anterior dimension; LVWPd, left ventricular wall posterior dimension; E-wave, early ventricular filling velocity; A-wave, ventricular filling velocity during atrial contraction; E/A, E-wave to A-wave ratio; ICT, isovolumetric contraction time; IRT, isovolumetric relaxation time; ET, ejection time; Tei Index, myocardial performance index = (ICT + IRT)/ET; LA area, left atrial area; PV-PA, peak velocity pulmonary artery; RV, right ventricle; RA area, right atrial area.

### Increased prenatal and postnatal mortality in Klf3^H275R^/+ and Klf3^H275R/H275R^ mutants

**Figure 4:**
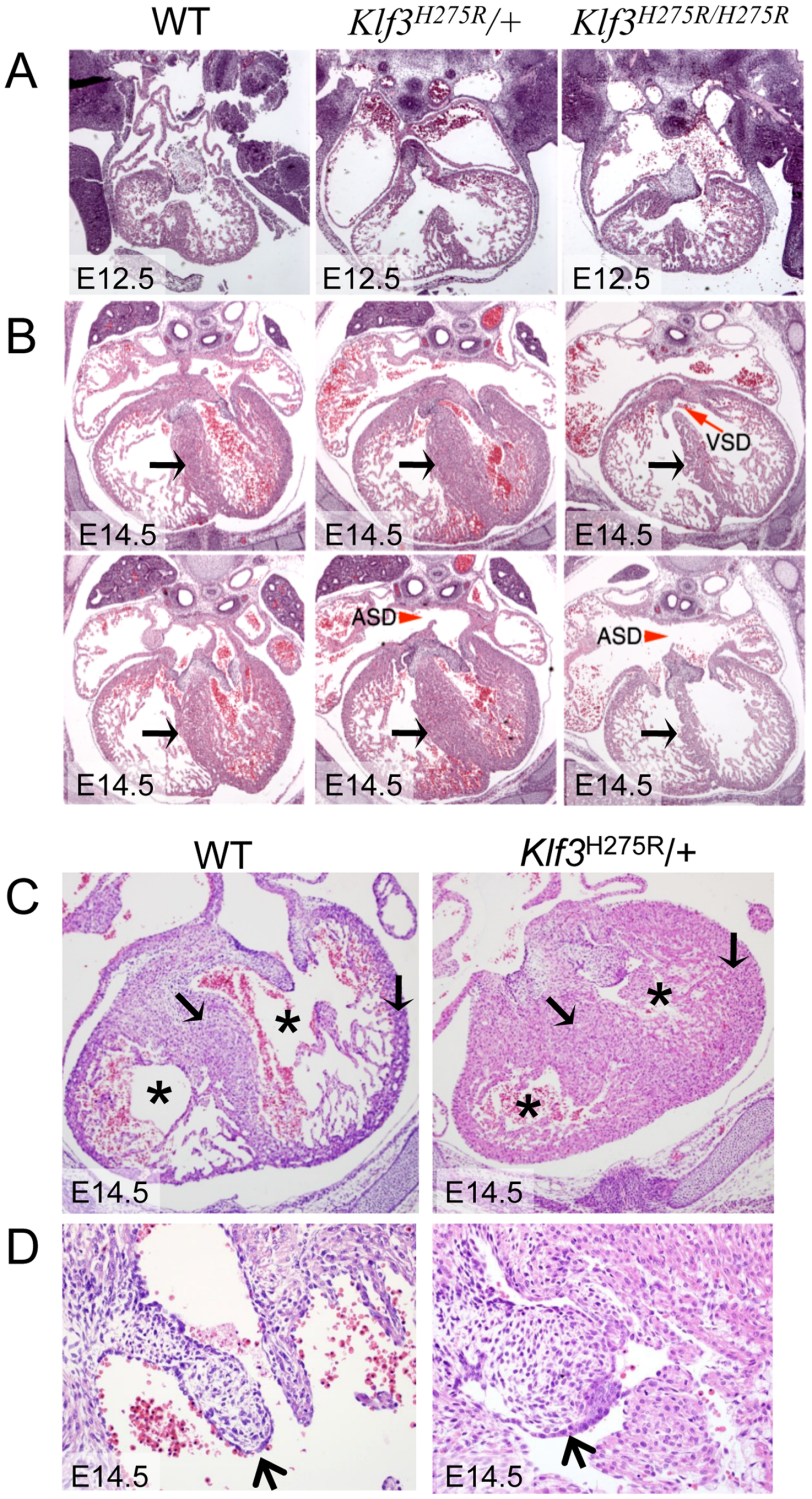
Abnormal cardiac histology in *Klf3^H275R^* embryos. In homozygotes, histological images showed thinned and disorganized ventricular myocardium and septum (A) at E12.5 and (B) in a rare homozygous survivor at E14.5 (two section levels from same specimen are shown). At E14.5, ventricular (VSD) and atrial septation defects (ASD) were observed in some homozygote and heterozygote hearts. (B) The interventricular septum of heterozygotes at E14.5 appeared thickened relative to WT and homozygotes (black arrows). (C) Heterozygous *Klf3* ^H275R^ hearts at E14.5 exhibited thickened myocardial and septal wall thicknesses (arrows), diminished left and right ventricular lumens (*), and (D) abnormal hyperplasia of atrioventricular cushion tissue (arrows) (enlargement from (C)). All homozygotes died prenatally. About half of the heterozygotes died before weaning.

**Figure 5:**
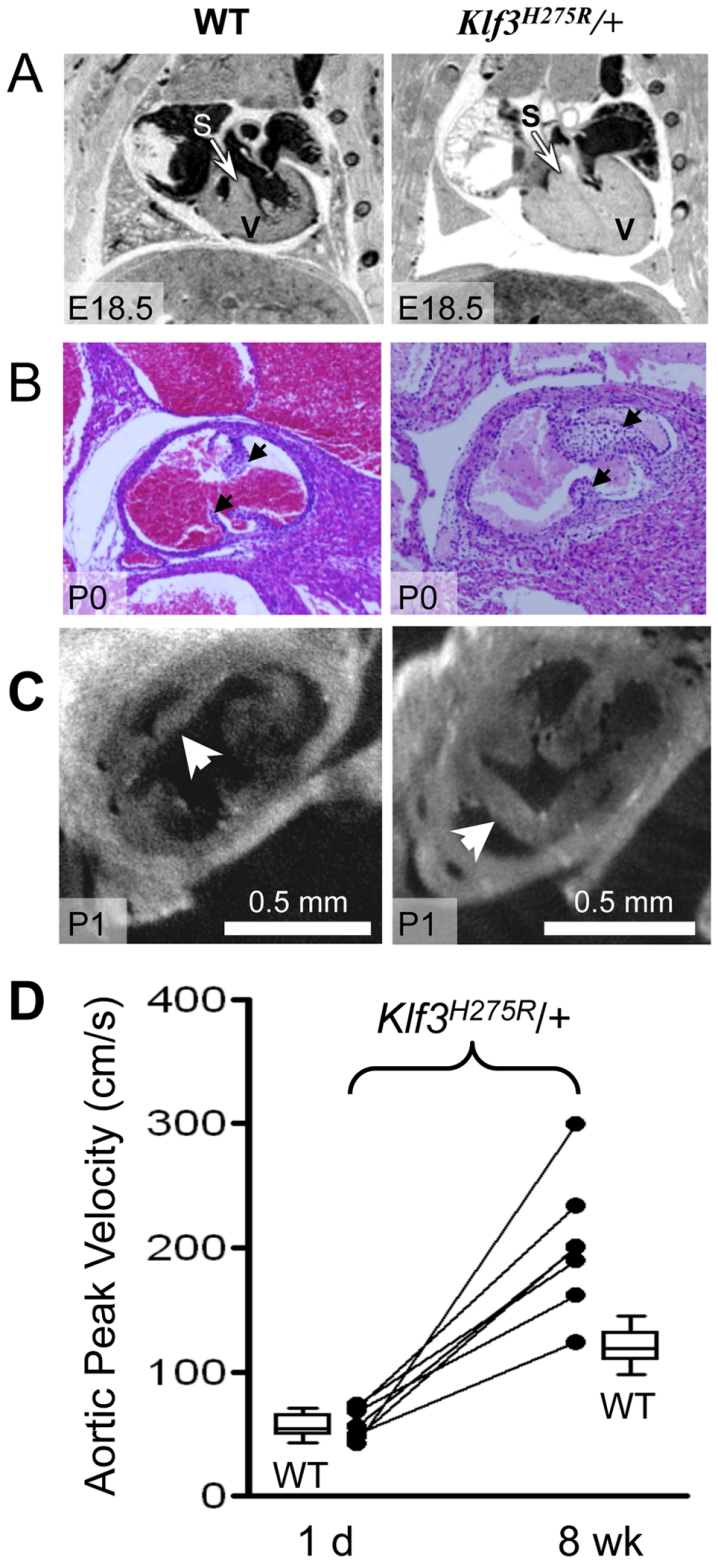
Abnormal hearts and aortic valves in *Klf3* ^H275R^/+ neonates. (**A**) Thoracic images of neonates delivered by cesarean-section at E18.5 (term). The heterozygous *Klf3* ^H275R^ neonate breathed occasionally for 30 min then stopped breathing and died (right). The wild type (WT) neonate (left) breathed and appeared normal before being euthanized for MRI. The ventricular myocardium (V) and septum (S) were markedly thickened in the heterozygous neonate that died. (**B**) Aortic valve leaflets (arrows) of a heterozygous *Klf3* ^H275R^ neonate found dead on postnatal day 0 (P0) were thickened (right) relative to a same age control (left). (C) Optical Projection Tomographic images of aortic valves from apparently healthy neonates on day 1 showing thickened aortic valve leaflets in *Klf3* ^H275R^/+ (right) compared to WT (left). (**D**) Serial measurements of aortic peak blood velocity in *Klf3^H275R^*/+ as neonates on day 1 and at 8 wk as adults (n = 7; solid lines join points). Box plots show WT values for day 1 neonates (n = 14) and 8–14 wk adults (n = 21). At day 1, all 7 *Klf3^H275R^*/+ mice had aortic peak velocities within the normal range whereas 6 of 7 were elevated by 8 wk.

At weaning, we observed 100% lethality of homozygous offspring and only ∼50% of the anticipated *Klf3* ^H275R^/+ pups from *Klf3* ^H275R^/+ intercross breeding (**Table S2**). At E14.5–16.5, significant lethality of homozygotes, but not heterozygotes, was observed (**Table S2**). We used histology to examine embryonic heart structure of *Klf3* ^H275R^ embryos at E12.5 (i.e. before the age of lethality) and at E14.5 in heterozygotes and in the few surviving homozygous embryos. At E12.5 (**Figure 4A**) and E14.5 (**Figure 4B**), homozygous embryos exhibited a thinned and disorganized ventricular myocardium and septum suggesting cardiac failure as the cause of their later demise. At E14.5, ventricular and atrial septation defects were also observed (**Figure 4B**). In contrast, heterozygotes at E12.5 had apparently normal cardiac anatomy (**Figure 4A**) and at E14.5 showed disorganization and *thickening* of the septal myocardium (**Figure 4B,C**). Some had atrial septation defects similar to homozygotes (**Figure 4B**) and enlarged atrioventricular cushion tissue that may have obstructed flow (**Figure 4C**). At birth, heterozygous neonates had abnormally thickened myocardial walls by magnetic resonance imaging (MRI) (**Figure 5A**) and aortic valve leaflets by histology (**Figure 5B**) and by optical projection tomography (**Figure 5C**). No abnormalities in placental weight or histology were detected at E12.5 and E14.5 (not shown) so placental dysfunction was unlikely to play a causative role (e.g. as in [21]).

To better define the age of lethality in heterozygotes, we delivered 3 litters of *Klf3* ^H275R^/+ crossed with B6 mice by caesarean section at term (E18.5). Three *Klf3* ^H275R^/+ embryos had recently died in utero and 2 *Klf3* ^H275R^/+ died within 30 min with only occasional breathing **(Table S2**). *Klf3* ^H275R^/+ pups that survived for up to 2 h were significantly smaller (1.02±0.02 g (n = 9)) than WT littermates (1.13±0.02 g (n = 10); P = 0.002). We next allowed 5 litters of *Klf3* ^H275R^/+ crossed with B6 mice to deliver naturally at term. One day after birth, cardiac hypertrophy in *Klf3* ^H275R^/+ pups was significant in surviving pups (7.5±0.2 mg/g body weight (n = 9) vs. WT 6.5±0.1 mg/g (n = 23); P <0.001) whereas it was striking in dead or dying pups on day 1 (15±2 mg/g (n = 3); P<0.001). Imaging showed that pups that died within 1 d of delivery had markedly diminished ventricular lumens, markedly thickened ventricular and septal myocardia, and aortic valve leaflets that were short and thick (**Figure 5A,B,C**). Thus, heterozygous *Klf3* ^H275R^/+ pups that had the most pronounced ventricular hypertrophy apparently died in the perinatal period.

### Cardiac hemodynamic dysfunction in postnatal Klf3^H275R^/+ mice

Aortic blood velocity was not higher in *Klf3* ^H275R^/+ pups at day 1 of age (49±4 cm/s; n = 9) relative to WT littermates (48±4 cm/s; n = 21). In *Klf3* ^H275R^/+ pups assessed on day 1 and again at 8 wk; 6 of 7 developed high velocities (>2 SD) by 8 wk (**Figure 5D**). Peak aortic blood velocity did not increase further between 9 wk and 1 y (n = 15; not shown). Thus, the *Klf3* mutation may alter prenatal aortic valve development but the development of sufficient stenosis to elevate aortic blood velocity is a postnatal event occurring by 8 wk in *Klf3* ^H275R^/+ mice.

Heterozygous mice that survived the perinatal period survived into adulthood. However the number of adults that died before 60 wk was significantly increased; 21% of *Klf3* ^H275R^/+ mice with high aortic blood velocities at 8 wk died under 60 wk of age (41 of 195) vs. 4% of WT cage-mates (2 of 54) (P<0.0001). Premature death in adulthood was associated with a rapid deterioration in health and all 4 mice found moribund exhibited marked cardiac enlargement (e.g. **Figure S3**; heart weight 0.367±0.040 g (n = 3) vs. 0.159±0.008 g (n = 3) WT cage mates). In the *Klf3* ^H275R^/+ group as a whole at ∼20 wk, cardiac enlargement was less pronounced and lung weight was not elevated (**Table S3**). Results are consistent with premature death caused by heart failure.

### Cardiac structure and function in adult heterozygous Klf3 point mutants

We anticipated that intraventricular pressures would be elevated due to aortic valvular stenosis in surviving adult *Klf3* ^H275R^/+ mutants and that this would lead to concentric ventricular hypertrophy (i.e. increased wall thickness) secondary to increased afterload. While the hearts were hypertrophic (i.e. the heart to body weight ratio was significantly elevated; **Table S3**), hypertrophy was not concentric because there was no increase in wall thickness in mutant adults (**Table 1**). Furthermore, the heart to body weight ratio did not correlate with aortic blood velocity (r^2^ = 0.17; P = 0.1; n = 13) or with the transvalvular pressure gradient (r^2^ = 0.06; P = 0.5; n = 7) in adult *Klf3* ^H275R^/+ mice. Indeed, left ventricular systolic blood pressure was not significantly elevated when directly measured in isoflurane-anesthetized mice (**Figure 2C**). Instead, we found that surviving adult *Klf3* ^H275R^/+ mice had eccentric hypertrophy (i.e. increased chamber dimensions). Thus, other prominent cardiovascular abnormalities were caused by the *Klf3* ^H275R^ allele; abnormalities not due to aortic valve defects.

Echocardiography on *Klf3* ^H275R^/+ mice was therefore performed to discover other effects of this mutation on adult cardiac function. We found that the diastolic volume of the left ventricle was significantly increased, and cardiac output was nearly doubled despite their smaller body weight (**Table 1**). The left and right atrial areas measured by ultrasound were also significantly increased in *Klf3* ^H275R^/+ hearts (**Table 1**), as was the heart to body weight ratio (**Table S3**). Left ventricular wall thicknesses and heart rate were unchanged (**Table 1**). *Klf3* ^H275R^/+ mice had improved systolic function as suggested by a significant increase in ejection fraction (**Table 1**) and improved diastolic filling as suggested by a 30% increase in the early filling (E-wave) velocity for left and right atrioventricular filling (**Table 1**). The high E-wave may explain its more frequent fusion with the A-wave in *Klf3* ^H275R^/+ mice (**Table 1**). Improved systolic and diastolic function was also supported by a change in the Tei Index (**Table 1**), a global indicator of cardiac function [22]. The change in the Tei Index occurred due to significantly shorter isovolumetric contraction and relaxation times, and a significantly longer ejection time (**Table 1**). Other than eccentric hypertrophy, there was no abnormality in histological structure of the heart, or atrial or ventricular myocardium detected by light microscopy (**Figure S4A**) or electron microscopy although myocardial contraction bands were more frequent in *Klf3* ^H275R^/+ mice (**Figure S4B**).

The *Klf3* ^H275R^/+ mutation caused a doubling of cardiac output (**Table 1**). Physiological increases in cardiac output can be evoked by increased metabolic rate or by reduced oxygen carrying capacity of the blood. Both would tend to increase tissue requirements for perfusion. We therefore measured oxygen consumption but found that it was only 15% higher in *Klf3* ^H275R^/+ mice (3112±28 ml h^−1^ kg^−1^ vs. 2709±22 ml h^−1 ^kg^−1^ in WT littermates, P = 0.03; n = 4 males per group averaged over 24 h) and thus was insufficient to explain the doubling in cardiac output in *Klf3* ^H275R^/+ mice. Similarly, although *Klf3* ^H275R^/+ mice were slightly anemic with a significant 10% reduction in RBC count (**Figure S5**) and haemoglobin concentration (not shown), reduced oxygen carrying capacity of the blood was insufficient to explain the large increase in cardiac output in these mutants.

### Arterial hypotension in adult heterozygous Klf3 point mutants

High cardiac output can also be induced physiologically by low total peripheral vascular resistance. This mechanism was implicated by the significant reduction in arterial blood pressure in *Klf3* ^H275R^/+ mice both when awake measured by tail cuff (98±2 mmHg vs. 108±1 mmHg in WT littermates at 9–12 wk; n = 10/genotype) and in the ascending aorta of anesthetised mice using a catheter-tip pressure transducer (**Figure 2C**). Heart rate did not significantly differ by genotype whether measured awake (677±13 min^−1^ *Klf3* ^H275R^/+ vs. 636±21 min^−1^ in WT; n = 10/genotype) or under anesthesia (**Table 1**). We found that low blood pressure was not caused by low plasma volume. Indeed, plasma volume measured by Evan's Blue dilution was 34% greater in *Klf3* ^H275R^/+ (44±3 ml/kg vs. 33±2 ml/kg in WT; P = 0.015; n = 6 per group). We were unable to find peripheral vascular malformations by color Doppler echocardiography, gross dissection, MRI or histology that could explain the low total peripheral vascular resistance.

These results suggest that the *Klf3* ^H275R^/+ mutation caused low peripheral vascular resistance by increasing peripheral vascularity and/or vascular calibre. The resulting low arterial pressure resulted in intraventricular pressures that were not elevated despite aortic valvular stenosis and this likely explains the absence of left ventricular wall thickening in adult *Klf3* ^H275R^/+ mutants. Thus the *Klf3* ^H275^ mutation caused other prominent cardiovascular abnormalities in addition to defects in aortic valve development.

### Body composition in heterozygous Klf3 point mutants

A defect in adipogenesis leading to reduced body weight and fat mass was the primary phenotype reported for homozygous mice with targeted deletion of the KLF3 zinc finger DNA binding domain [12]. An *in vitro* role for KLF3 in adipocyte differentiation was also found. In young adult *Klf3* ^H275R^/+ mutants in our study, percent body fat was reduced by 14% (P<0.04; n = 13/genotype) and body weight by 8% (P<0.001; n = 13/genotype) in surviving *Klf3* ^H275R^/+ mice at 10–11 wk. At 18–25 wk, the relative weight of the superficial abdominal fat pad was reduced by 43%, which contrasted with increased relative weights of the heart, spleen, kidney, and brain (**Table S3**). Relative lung and liver weights were not affected (**Table S3**). The similarity in body weight and fat mass phenotype between *Klf3* ^H275R^/+ and *Klf3* DNA binding domain deletion mutants [12] suggested that *Klf3* ^H275R^ is a loss of function allele. However, divergent traits (below) suggest that the interaction is more complex.

### Effect of Klf3 point mutation on Klf3 mRNA and protein function

**Figure 6:**
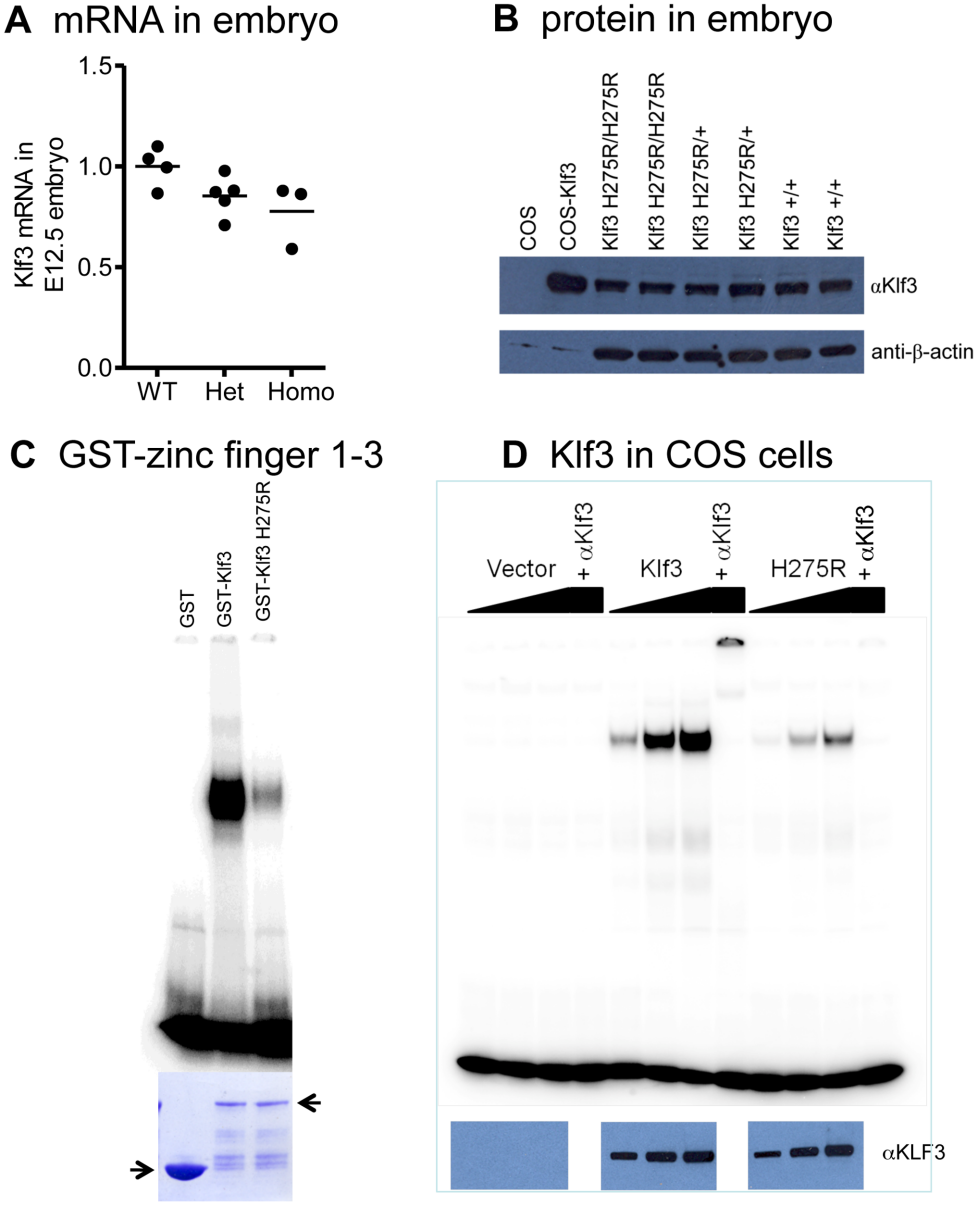
*Klf3^H275R^* mRNA, protein, and impairment in DNA binding. (A) Similar levels of *Klf3* mRNA by qRT-PCR (P>0.05) and (B) KLF3 protein by Western blot (WB) in whole H275R homozygotes (Homo) and heterozygotes (Het) versus wild-type (WT) embryos at E12.5. (C) Bacterial expression of GST-zinc finger 1–3 of normal and mutant protein showing that H275R impaired DNA binding to CCACACCCT (canonical β-globin promoter) in electrophoretic mobility shift assay (coomassie blue-stained SDS PAGE gel (below)). (D) Dose-response showing H275R also impaired binding to CCACACCT in electrophoretic mobility shift assays when full length *Klf3* was expressed in COS cells (western blot with αKlf3 antibody below). Protein identity was validated by eliminating binding with a KLF3 antibody (αKlf3).

*Klf3* mRNA (**Figure 6A**) and protein (**Figure 6B**) were expressed at wild-type levels in homozygous and heterozygous *Klf3* ^H275R^ embryos at E12.5. H275R protein exhibited reduced binding to KLF3's canonical CACCC binding region of the β-globin gene promoter both using recombinant bacterial GST-*Klf3* zinc finger 1–3 protein (**Figure 6C**) or full length KLF3 protein expressed in COS cells (**Figure 6D**), but did not interfere with the ability of WT KLF3 to bind to DNA (**Figure S6**). *In vivo*, the H275R protein significantly opposed the repression of *Lgals3*, a gene that is normally repressed by KLF3 [14], in *Klf3* ^H275R^ homozygous embryos at E12.5 (**Figure S7**). However, expression of other known KLF3 targets including *Klf8*, *Crip1*, and *Pqlc3* (Crossley M et al. unpublished) was unaffected (not shown).

### Klf3 deficiency causes abnormal cardiovascular development in zebrafish and mice

**Figure 7:**
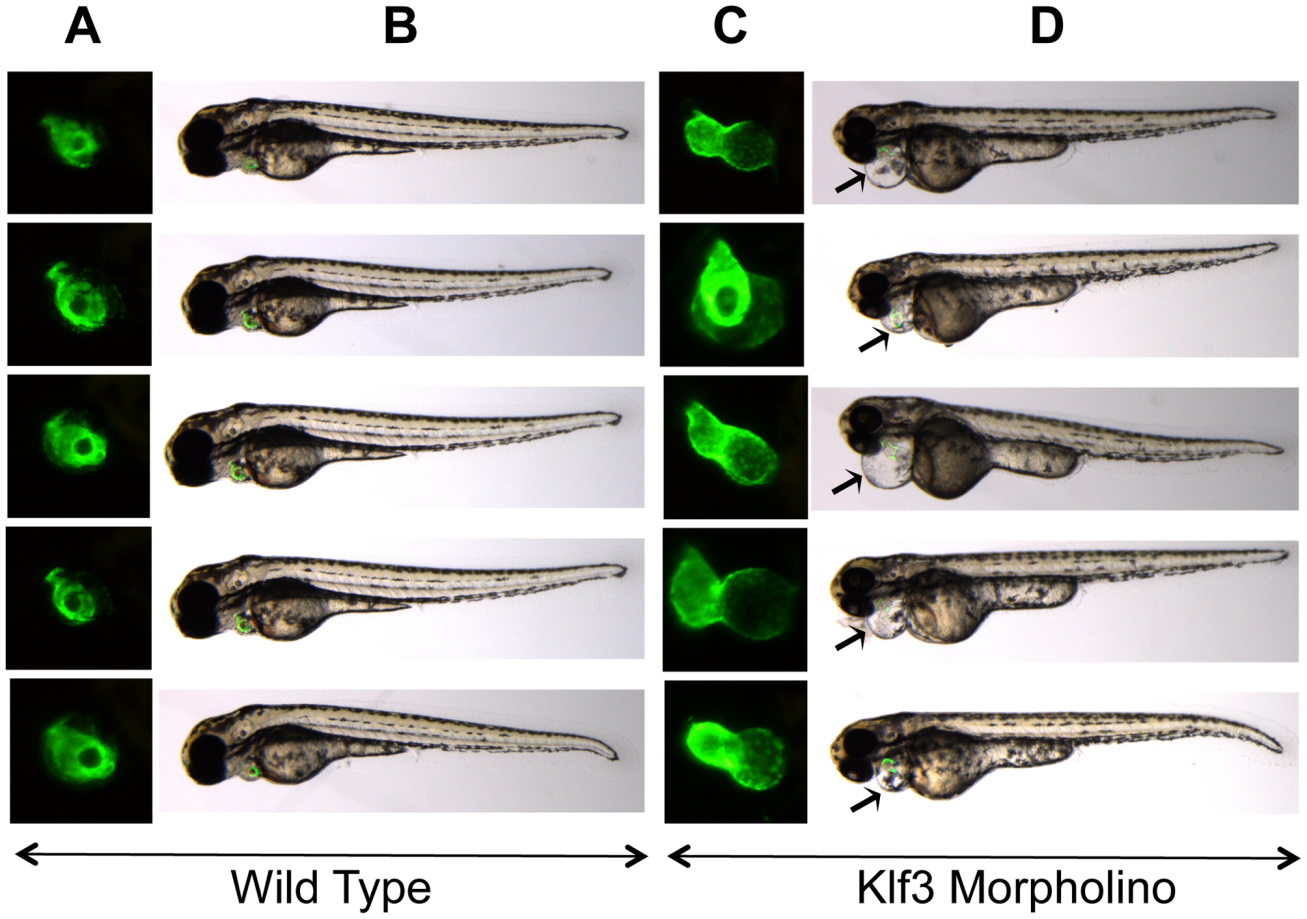
Cardiac edema in zebrafish embryos injected with *klf3* morpholinos. (A,C) Enlarged GFP images of embryonic hearts at 72 hpf. GFP expression was driven by the *myl7* regulatory element (cardiac myosin light chain 2) and marks the myocardium of the ventricle (rostral) and atrium (caudal). (B,D) Light microscopy images of the corresponding zebrafish embryos with the GFP heart image shown in (A,C) superimposed. The GFP heart is in the same orientation as in (A) but has not yet been enlarged. Most embryos injected with *klf3* morpholino exhibited cardiac abnormalities as shown in (C) and cardiac edema shown by arrows in (D) in comparison to wild type embryos shown in (A,B). Injection of the mismatch control morpholino had no effect on the developing zebrafish heart (not shown).

Because a role for KLF3 in cardiovascular development and function was hitherto unknown, we asked whether reduced KLF3 function could cause abnormal cardiovascular development in zebrafish embryos by using anti-*klf3* morpholinos to inhibit translation of *klf3* transcript. At 48 hour post-fertilization (hpf), embryos appeared to be developing normally suggesting that initial differentiation and morphogenesis of the heart proceeded normally. However by 72 hpf, 65% of *klf3* knockdown embryos exhibited cardiac edema indicative of cardiovascular dysfunction (i.e. 49 of 59, and 27 of 58 in 2 replicate experiments) and some hearts were visibly dysmorphic and did not properly loop (**Figure 7**). Of those with edema, blood flow was visible in the embryonic vasculature in 60% of embryos at 72 hpf (i.e. 22 of 49, and 8 of 27 in the 2 replicates) with no occlusion of the outflow tract evident (data not shown). This supports cardiac dysfunction as the primary cause of cardiac edema and heart defects. Results were consistent over 6 replicate experiments (50–100 embryos per experiment). Injection of the mismatch control morpholino had no effect on the developing heart.

### Lethality and the cardiovascular phenotype of Klf3 gene trap mutants

**Figure 8:**
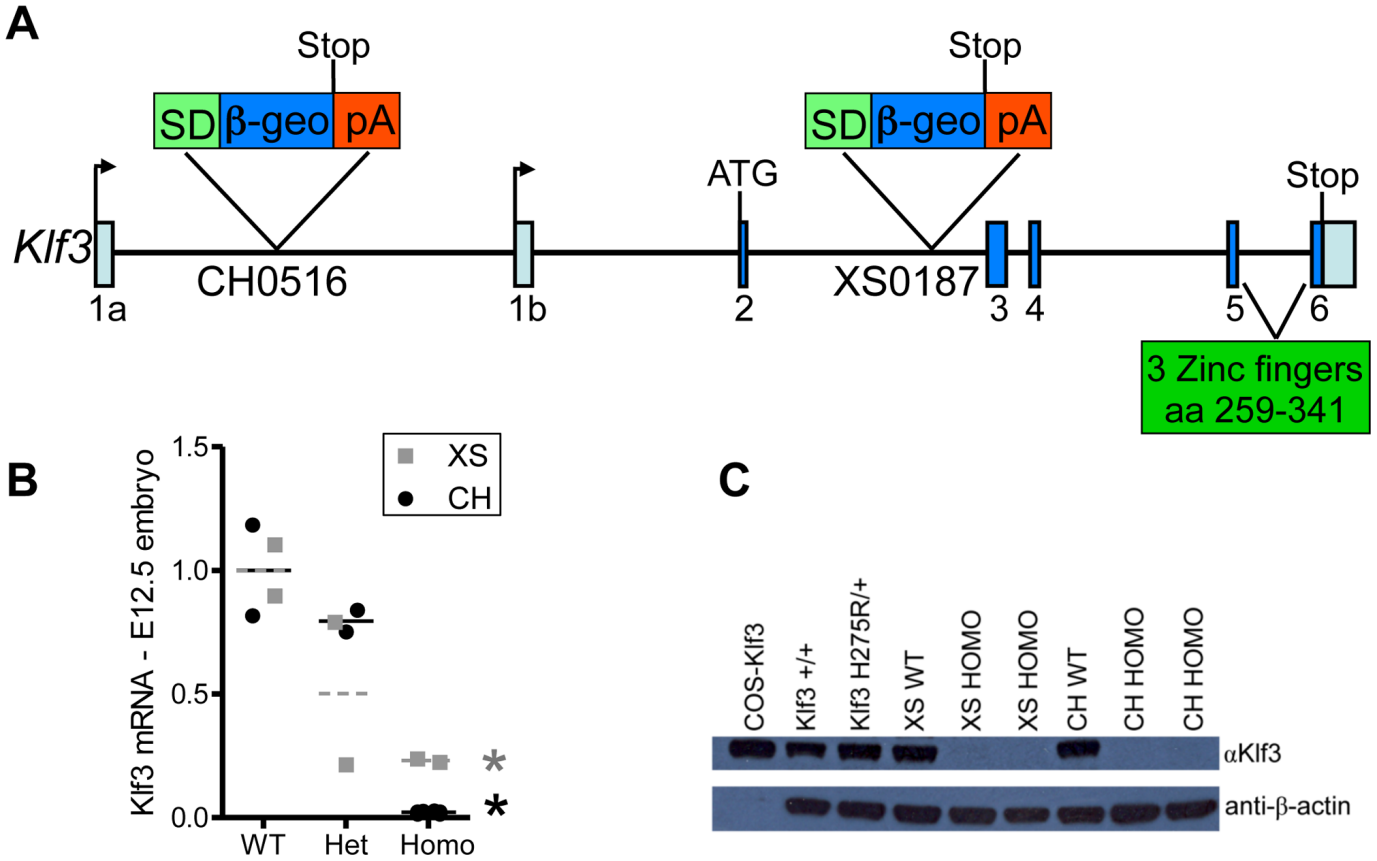
Gene trap insertion sites and effects on *Klf3* mRNA and protein. (A) Schematic of *Klf3* gene showing the DNA insertion site of the gene trap marker in the XS line (XS0187) and in the CH line (CH0516). SD = splice donor site; β-geo = β-galactosidase and neomycin fusion protein; pA = polyadenylation. (B) *Klf3* mRNA in E12.5 whole embryos from the XS (grey squares) and CH (black circles) gene trap lines measured by qRT-PCR was significantly decreased in homozygotes (homo) but not heterozygotes (het). (C) KLF3 protein in spleens of Klf3^H275R^/+ adults was similar to WT (i.e. Klf3+/+, XS WT, CH WT) whereas it was undetectable in XS and CH homozygote spleens by western blot.

To further validate that altered KLF3 function was the cause of the cardiovascular defects observed in *Klf3* ^H275R^ mutants, we generated mutant mice from two embryonic stem cell lines with gene trap vectors inserted near the start of the *Klf3* gene (XS and CH; **Figure 8A**). These insertions largely eliminated *Klf3* mRNA (**Figure 8B**) and KLF3 protein (**Figure 8C**) expression in homozygotes. If *Klf3* ^H275R^ was a simple loss of function allele, then these gene trap mutants would be anticipated to exhibit perinatal lethality and cardiovascular defects similar to *Klf3* ^H275R^ mutant mice.

At weaning, heterozygosity did not affect survival in *Klf3* gene trap mutants. This contrasted with ∼50% of *Klf3* ^H275R^ heterozygotes dying in the perinatal period. However, there was significant lethality prior to weaning age in homozygous gene trap mutants compared to WT littermates (**Table S4**). This was reported previously for homozygous mutants lacking *Klf3*'s DNA binding region where ∼half those anticipated were found at weaning whereas the anticipated ratio was observed at E14.5 [12]). We found that some homozygous gene trap mutants survived to adulthood whereas homozygosity of the point mutation was always embryonic lethal.

**Figure 9:**
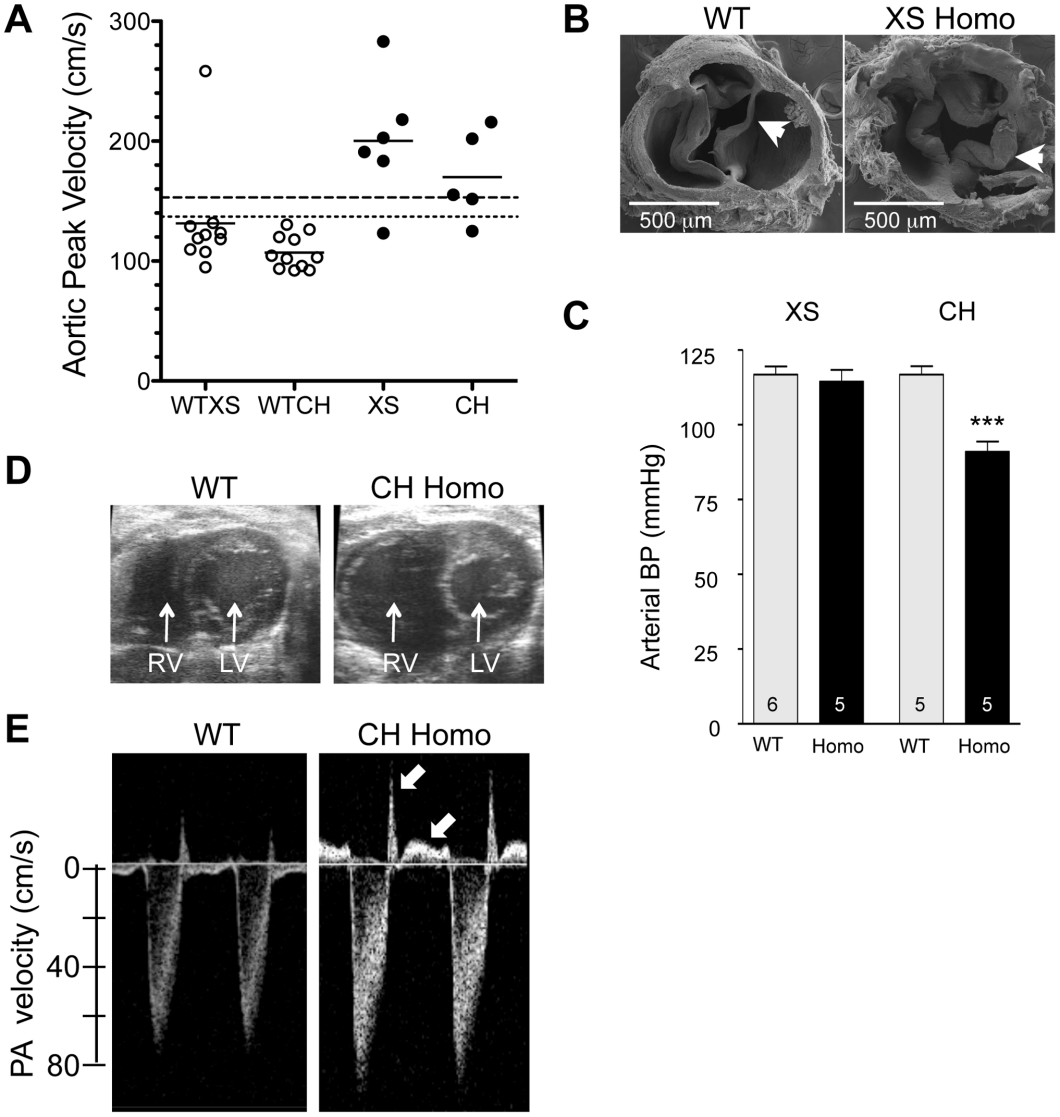
Cardiovascular changes in adult homozygous (homo) gene deletion mutants. (A) Elevated peak blood velocity in the ascending aorta of XS and CH gene trap mutants (solid circles) relative to WT controls (open circles). Group means are indicated by horizontal lines. The lower dashed line shows the mean + 2SD and the upper dashed line the mean + 3SD for WT point mutant controls. 5 of 6 XS, and 4 of 5 CH mice had elevated peak velocities (>2SD relative to WT point mutants). (B) Scanning electron microscopy images of the aortic valve of a WT littermate (left) and a XS homozygote with aortic valvular stenosis (right). Note marked thickening of the valve leaflets of the mutant. (C) Arterial blood pressure by tail cuff plethysmography in XS and CH lines. *** P<0.001. N is shown in the bar. Mean ± SE. Note that significant hypotension was observed in the CH line only. (D) Cross-sectional view of the heart of a WT and CH homozygous mutant. Note the enlarged right ventricle (RV) in the mutant. Prominent RV enlargement was observed in 2 of 7 XS and 6 of 8 CH homozygotes but no WT controls. (E) Doppler velocity waveform in the proximal pulmonary artery in a WT and CH homozygous deletion mutant. Reversed diastolic blood velocities (arrows) indicate pulmonary valve regurgitation (observed in 5 of 7 XS and 8 of 8 CH homozygotes but no WT).

To evaluate the effect of *Klf3* gene trap mutations on the aortic valve, we measured ascending aortic blood velocity in homozygous adults. We found that aortic velocity was elevated in a significantly greater proportion of surviving homozygotes of both gene trap lines (**Figure 9A**). Heterozygotes were not significantly affected (not shown). Homozygote gene trap mutants often exhibited thickened aortic valve leaflets by gross morphology (5 of 7 mutants vs. 0 of 6 WT littermates) (e.g. **Figure 9B**). Thus, homozygous *Klf3* gene trap mutations caused stenotic, malformed aortic semilunar valves that resembled those of the heterozygous point mutant mice (*Klf3* ^H275R^/+).

Like adult *Klf3* ^H275R^ heterozygotes, homozygous gene trap mutants also had other diverse cardiac defects. They had significantly higher cardiac output (**Table 1**) with no change in left ventricular wall thickness or heart rate (**Table 1**) and, in homozygous CH mice, significantly higher left ventricular end diastolic volume (**Table 1**), consistent with a phenotype of left ventricular eccentric hypertrophy. Homozygous CH mice also had significantly reduced arterial pressure (−26 mmHg) (**Figure 9C**), which was similar to *Klf3* ^H275R^ heterozygotes (−10 mmHg) (**Figure 2C**). Also like adult *Klf3* ^H275R^ heterozygotes, homozygous gene trap mutants had significantly reduced body weights, enlarged hearts, and decreased abdominal fat pad weights (**Table S5**).

Homozygous *Klf3* gene trap mutants exhibited a pronounced right ventricular trait not observed in *Klf3* ^H275R^/+ mice. They often had marked pathological enlargement of the right ventricle (8 of 15; e.g. **Figure 9D**) and an abnormal leftward septal deviation in early diastole (M**ovie S1** **and S2**). Many also exhibited abnormally thickened pulmonary valve leaflets (**Figure S2C,D**), and pulmonary valve regurgitation (13 of 15) that was often associated with tricuspid valve regurgitation (7 of 15) (**Figure 9E**). Lung weight was significantly increased (**Table S5**) consistent with pulmonary congestion. No septal defects were detected. It is noteworthy that valve closure and septal deviation abnormalities could be secondary to a primary right ventricular enlargement defect.

There were also differences in the haematological phenotypes of the point mutant and gene trap mutants. All 3 mutant lines exhibited greater variation in red cell volume (%RDW; **Figure S5**) and a larger number of reticulocytes (immature RBC) in blood smears suggesting a defect in erythrocyte production, structure, and/or elimination. However, only *Klf3* ^H275R^ heterozygotes were anaemic and had increased RBC cell size, and only homozygous gene trap mutants had increased white blood cell counts (WBC) (**Figure S5**).

In *Klf3* ^H275R^/+ mice, slight anaemia was deemed insufficient to explain the large increase in cardiac output in these mutants. This is supported by the finding that homozygous gene trap mutants had increased cardiac output (**Table 1**) but no significant change in RBC count (**Figure S5**) or haemoglobin concentration (not shown).

### Organ distribution of Klf3 expression

**Figure 10:**
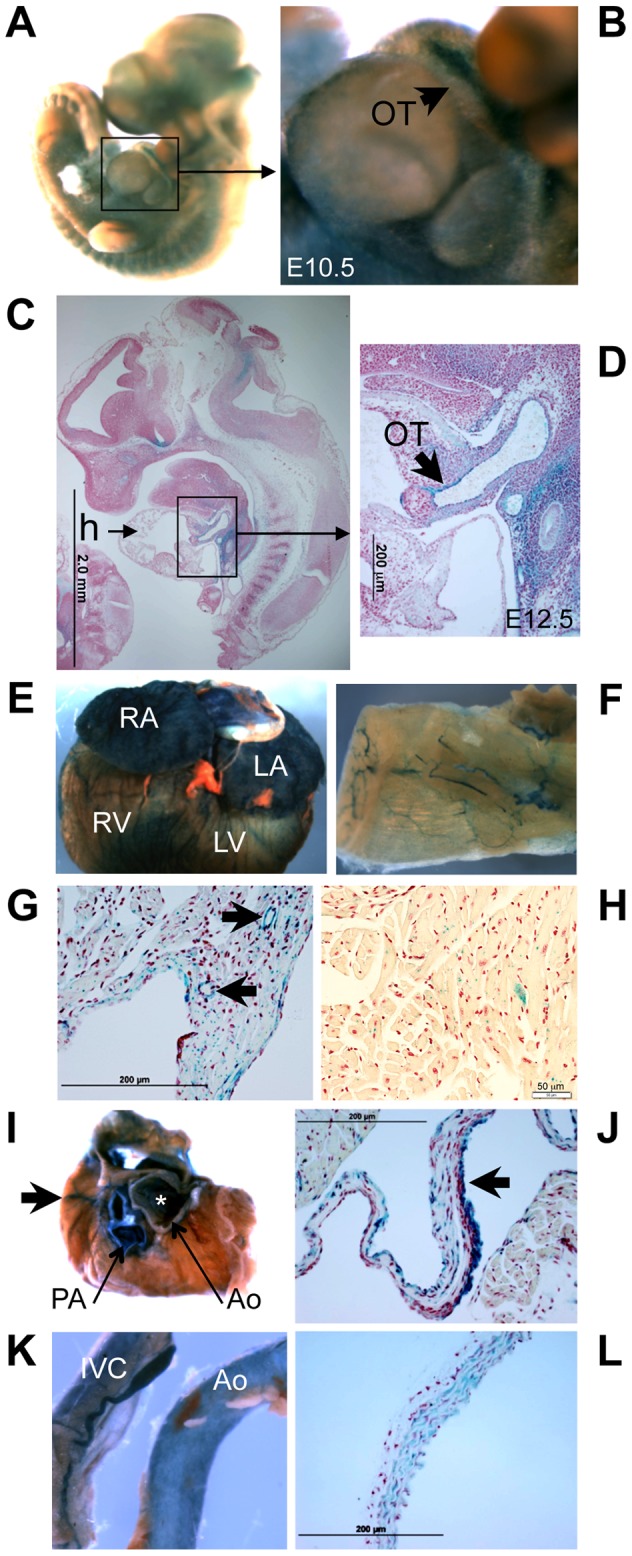
*Klf3* gene expression detected by LacZ staining in the developing and adult heart and vasculature. LacZ -staining (blue) in homozygous XS mice shows *Klf3* gene expression is located at discrete but widespread sites in (A) the E10.5 embryo (whole mount) including (B) the embryonic outflow tract (OT), and (C) in histological sections of E12.5 embryos particularly in (D) the outflow tract. In adult XS mice, prominent *Klf3* gene expression was observed in (E) the atria and ventricles and in (F) blood vessels including those of the skeletal muscle shown here in LacZ stained tissues. Histological images show *Klf3* expression in the myocardium of the (G) atrium (where blood vessels are also heavily stained (arrows)) and in (H) the ventricle where staining is diffuse and punctate. (I) After removing the atria from the heart, prominent expression was seen in cardiac valves (asterisk indicates aortic valve location) and in the aorta (Ao) and pulmonary artery (PA), and coronary vessels (arrows) of the heart. Histological images show strong staining in (J) the aortic valve leaflets (arrow). *Klf3* gene expression was prominent in (K) the inferior vena cava (left) and aorta (right) and in (L) histological images of vasculature where staining was found in both the smooth muscle and endothelium as shown here for the aorta. Ao, aorta; h, heart; IVC, inferior vena cava; LA, left atrium; LV, left ventricle; OT, outflow tract; PA, pulmonary artery; RA, right atrium; RV, right ventricle.

Staining for *LacZ* generated by the XS *Klf3* gene trap vector indicated strong *Klf3* expression in the E10.5–12.5 embryonic aorta and cardiac outflow tract (where heart valve primordia form) (**Figure 10A–D**). LacZ staining in the embryonic myocardium was diffuse and punctate (not shown). LacZ staining was also observed in the adult atrial and ventricular myocardium, heart valves, and endothelial and vascular smooth muscle of the vasculature (**Figure 10E–L** **, Figure S8**). Thus local alterations in KLF3 transcriptional activity in cardiovascular cells may directly cause aortic valvular stenosis, hypotension, and abnormal myocardial growth. Strong *LacZ* staining was observed at other discrete albeit widespread sites within the embryo (e.g. **Figure 10A,C**) as observed previously by ISH in embryos [8], [23] and by qRT-PCR in adult tissues [9] so indirect hormonal or neural mechanisms may also play a role in cardiovascular abnormalities in *Klf3* mutants.

### Altered embryonic gene expression caused by *Klf3* ^H275R^ and CH *Klf3* gene trap mutations

Gene expression was evaluated in *Klf3* ^H275R^ and CH homozygotes, *Klf3* ^H275R^ heterozygotes, and WT by microarray (**Figure S9**). To increase the likelihood of revealing immediate downstream targets of KLF3, we used mRNA isolated from whole embryos collected at E12.5 d of gestation. At this stage, *Klf3* ^H275R^ homozygosity was not yet lethal, and *Klf3* LacZ expression was widespread (e.g. **Figure 10A**). When comparing *Klf3* ^H275R^ homozygotes, heterozygotes, or both groups combined versus WT, no genes were identified as significantly changed using a false discovery rate threshold of 0.1. For CH homozygotes vs. WT, 18 genes were changed significantly and 2 were changed >2-fold; *Klf3* (0.07×) and *Hsd3b6* (0.23×) (**Table S6**). Overall, CH homozygous embryos had 16 genes differentially expressed when compared to *Klf3* ^H275R^ homozygotes (including 4 genes changed by >2-fold; *Klf3* (0.07×), *Glmap4* (2.07×), *Snora31* (2.20×), and *Fah* (2.31×)) and 185 genes were differentially expressed when compared to *Klf3* ^H275R^ heterozygotes (including 5 genes changed by >2-fold; *Klf3* (0.07×), *Hsd3b6* (0.38×), *Mir1948* (0.48×), *Snora31* (2.04×), and *Slc6a16* (2.24×)) (**Table S6**). These differences in embryonic gene expression profiles may explain phenotype differences between heterozygous point mutant and homozygous gene trap lines that were observed later in development.

We then used qRT-PCR to validate the large down regulation of *Hsd3b6* expression found in E12.5 embryos by microarray. Expression was significantly reduced in *Klf3* ^H275R^ heterozygotes (0.45×), and in *Klf3* ^H275R^ homozygotes (0.27×) vs. WT (**Figure S10A**) whereas a similar trend in CH homozygotes was not statistically significant possibly due to the smaller sample size (**Figure S10B**). We also evaluated *Lilra6* by qRT-PCR; among genes with apparent up-regulation by visual inspection of the heat map (**Figure S9**), *Lilra6* was the most consistently high in *Klf3* mutant embryos (8 of 11) and low in WT (4 of 4). *Lilra6* was significantly elevated in *Klf3* ^H275R^ heterozygotes (2.4×), and in *Klf3* ^H275R^ homozygotes (13.8×) (**Figure S10C**) whereas the much smaller increasing trend in CH homozygotes (2.2×) was not statistically significant (**Figure S10D**). These results show that the heterozygous and homozygous presence of the point mutant protein can down or up regulate normal gene expression in E12.5 embryos, and that effects may differ from that caused by reduced expression of the native protein (i.e. in CH homozygotes).

## Discussion

Herein we report the discovery of novel and important roles for *Klf3* in cardiovascular development and function. Although KLF3 was identified and cloned nearly 20 years ago [8] and *Klf3* knock out mice have been studied [12], KLF3's role in cardiovascular biology had not been revealed until the current unbiased genome-wide ENU screen, in which prominent abnormalities were discovered in heterozygous *Klf3^H275R^* point mutants. We found abnormalities in embryonic gene expression during organogenesis and in heart morphology at E12.5, increased perinatal lethality associated with marked biventricular myocardial hypertrophy and aortic valve leaflet thickening, and adult survivors exhibited hypotension, aortic valvular stenosis, aortic dilatation, and myocardial hypertrophy with increased chamber size. KLF3 *in vitro* is enriched at promoters of muscle-specific genes [11], and *in vivo* is expressed by multiple cell types integral to the cardiovascular system including cardiomyocytes, heart valves, and vascular endothelial and smooth muscle cells based on LacZ expression patterns reported here. Cardiovascular abnormalities may therefore result directly from abnormal KLF3 function in cardiac and/or vascular cells. Abnormal renal or brain regulatory mechanisms may also contribute to hypotension given that *Klf3* is expressed at these sites as well (e.g. figure 9 A,B; ref #10). Elucidating the likely multifactorial roles of KLF3 in cardiovascular development and function will require temporal- and cell-type specific control of *Klf3* expression in future studies.

A role for KLF3 in cardiac valve development was detected in our ENU mutagenesis screen, in which high aortic blood velocity revealed aortic semilunar valve stenosis in heterozygous adults with *Klf3* ^H275R^ point mutations. In contrast, other cardiac valves were relatively unaffected although they similarly expressed *Klf3* based on LacZ staining. Valve specificity may arise due to differences in developmental mechanisms in semilunar versus atrioventricular valves [24]. For example cells derived from the secondary heart field [25] and cardiac neural crest [26] selectively contribute only to semilunar valve formation. Homozygous gene trap mutants also exhibited a high incidence of aortic valvular stenosis in adults but, in contrast with point mutants, the pulmonary semilunar valve leaflets sometimes appeared thickened histologically and often failed to close sufficiently to prevent regurgitation. In gene trap mutants, it is possible that pulmonary valve defects were secondary to a primary chamber enlargement defect of the right ventricle, a trait that was also never observed in heterozygous *Klf3* ^H275R^ point mutants. Although aortic valve cusps were thickened in late gestation in *Klf3* ^H275R^/+ embryos, aortic valvular stenosis sufficient to elevate aortic blood velocity developed only after birth when the normal separation, elongation, and thinning of the valve leaflets occurs [27], [28]. This suggests KLF3 plays a particularly critical role in these later events in aortic valve maturation. This finding is especially interesting given the paucity of knowledge about the genetic regulation of aortic semilunar valve development and the prevalence of aortic valve defects in humans [24].

Additional phenotypic characterization revealed broader roles for KLF3 in cardiovascular development and function, roles that were independent of its role in aortic valve development. In adults, high cardiac output and cardiac hypertrophy due to chamber enlargement (i.e. eccentric growth) with no change in left ventricular wall thickness was paradoxical given aortic valvular stenosis in adult *Klf3* point mutant and gene trap mutants. This was explained by the surprising observation that stenosis did not elevate intraventricular systolic blood pressure in *Klf3^H275R^*/+ hearts relative to WT. Instead, normal intraventricular pressure, low arterial blood pressure, and augmented blood volume in adult *Klf3^H275R^*/+ were likely caused by low systemic vascular resistance, resulting in a hyperdynamic circulation and the high cardiac output that we observed. Intriguingly, hypotension in a wide variety of other mouse models does not elicit cardiac hypertrophy and/or an increase in cardiac output. Examples include transgenic mice with overexpression of eNOS (−18 mmHg and no change in heart weight to body weight ratio) [29], Rgs5-deficiency (>−20 mmHg and no increase in left ventricular inner diameter in diastole) [30], and overexpression of atrial natriuretic factor (−24 mmHg and no change in cardiac output) [31]. One exception is homozygous deletion of *sarcomeric mitochondrial creatine kinase* (*Ckmt2*) where hypotension, left ventricular hypertrophy and high cardiac output can occur depending on genetic background [32], [33]. Interestingly, KLF3 acts as a transcriptional activator of muscle creatine kinase [11] and interacts with promoters of several other muscle-specific genes but whether it influences *Ckmt2* expression is not known. Thus, although a hyperdynamic circulation may cause eccentric cardiac growth, a direct myocardial hypertrophic mechanism may also be involved.

In the embryo at E12.5, prominent *Klf3* LacZ expression was present in the ventricular outflow tract and vascular endothelium whereas LacZ expression in the myocardium was diffuse and punctate. It is therefore possible that the thin myocardium in *Klf3^H275R^* homozygote embryos was secondary to a vascular defect, which caused low peripheral vascular resistance and low intracardiac pressures. A thin myocardium also occurs by E12.5 in mouse embryos with endothelial-specific *Klf2* gene deletion [34]. In that model, phenylephrine (a vasoconstrictor) reduced lethality at E14.5 in mutant mouse embryos, and in zebrafish embryos injected with anti-*klf2* morpholino [34]. However, when we injected zebrafish with anti-*klf3* morpholino, phenylephrine failed to significantly reduce lethality (not shown). Also we observed cardiac septal defects whereas none were reported in *Klf2* endothelial-specific knockout mouse embryos [34]. Thus, at E12.5, cardiac defects were likely caused by the direct myocardial effects of *Klf3* ^H275R^ protein although contribution from vascular defects cannot be ruled out. Later in gestation, LacZ staining appeared to become more prominent in the heart and heart valves, ventricular wall thicknesses and heart weights were increased, ventricular chamber sizes were diminished, and aortic valves appeared abnormally thickened. Pressure loading of the heart due to outflow tract obstruction may have contributed to cardiac hypertrophy, which was dramatic in mutant embryos that died in the perinatal period. However, in surviving newborns, aortic blood velocities were not significantly elevated suggesting minimal aortic valvular stenosis. Nevertheless, left ventricles were significantly hypertrophied. These data suggest that at least a component of the perinatal hypertrophic cardiac phenotype was a direct effect of *Klf3* ^H275R^ on cardiac development and growth.

When we compared the phenotype of the H275R mutation with the loss of function gene trap mutations in *Klf3*, we found that there were considerable similarities, but also marked differences, between the heterozygous point mutants and the homozygous gene trap mutants. Similarities may be explained by the point mutation acting as a dominant negative so that it interfered with wild-type KLF3 function. However, the more severe embryonic lethality shown by the homozygous point mutants than the homozygous gene trap mutants, suggests that the point mutant form of KLF3 may also disrupt other proteins and pathways. It is not uncommon for point mutants to display phenotypes that are more robust and/or different than null mutants for the same gene [35] because point mutations can cause not only reduced gene function, but also enhanced or abnormal gene function, and/or generate proteins with dominant negative activity. Some phenotype differences between *Klf3* ^H275R^ mutants and *Klf3* gene trap mutants may be due to differences in genetic background; phenotyping of *Klf3* ^H275R^ was largely on a B6 background whereas *Klf3* gene trap mutants were on a mixed 129/B6 background. However, genetic background within the *Klf3* ^H275R^ strain cannot account for the more severe phenotype in homozygotes (lethal by ∼E14.5–16.5) than heterozygotes (∼50% survived to adulthood). Thus results are inconsistent with a simple dominant negative effect of the mutant protein. This is also supported by our finding that the mutant *Klf3* ^H275R^ protein exerted a dominant negative action on one of KLF3's gene targets (*Lgals3*) while sparing other normal targets. In this case, it is not clear how the point mutant protein would disable the native protein to exert a dominant negative effect because KLFs are not known to dimerize [36]. Furthermore, the point mutant protein did not interfere with the ability of WT KLF3 to bind to KLF3's canonical CACCC DNA binding region *in vitro*. Nevertheless, *in vivo*, the point mutant protein may have a greater affinity for rate-limiting co-factor binding proteins thereby competitively inhibiting the activity of native protein. In addition, alterations in the mutant protein's DNA binding specificity may cause the mutant protein to transcriptionally regulate additional genes not normally regulated by KLF3 (i.e. a gain of function effect).

A similarly complicated interaction caused by a single amino acid change in the second zinc finger of *Klf1* (E339D) has also been found in mutant mice [36], [37], and the homologous mutation in humans causes human disease [38]. Similar to our case, the *Klf1* mutation changed the central of 3 amino acids predicted to contact DNA in the zinc finger of a KLF protein. That mutation resulted in a mutant KLF1 protein that failed to bind and transactivate a subset of KLF1's downstream targets in a manner dependent on the target gene's DNA binding sequence [36]. While an association between the *Klf3* ^H275R^ mutation and human disease is not currently known, autosomal dominant point mutations, like H275R, are among the most common causes of human genetic disorders. With *Klf1* as precedent, the current work provides strong impetus for searching for mutations in *Klf3* in humans with cardiovascular dysfunction.

In conclusion, we have discovered important and hitherto unknown roles for *Klf3* in cardiovascular development and function. We have also revealed the critical importance of amino acid 275 in the DNA binding region of the first of three zinc fingers, for normal DNA binding and transcriptional gene regulation, and its importance in cardiovascular development. This histidine residue is conserved in KLF3 across species from zebrafish to humans, and in all but one of 22 Sp/Klf family members [18], [28] (http://www.ncbi.nlm.nih.gov/sites/entrez?cmd=Retrieve&db=homologene&dopt=MultipleAlignment&list_uids=7). Thus the identification of the critical importance of this histidine residue may have broad implications; it may apply across species, across the Sp/Klf family, and across other C2H2 zinc finger (C2H2-ZNF) proteins, one of the largest and most complex gene super-families with hundreds of members in the human and mouse genome [29]. Involvement of this histidine residue within the zinc finger domain in human disease warrants further investigation.

## Materials and Methods

### Ethics statement

All experimental procedures received approval from the Animal Care Committee of Mount Sinai Hospital and were conducted in accordance with the guidelines of the Canadian Council on Animal Care.

### Generation of ENU mutant by Centre for Modeling Human Disease (www.cmhd.ca)

When male C57BL/6J mice (www.jax.org) regained fertility after ENU treatment (85 mg/kg i.p. 1/wk for 3 wk), they were bred to C3H/HeJ females (www.jax.org). Offspring were screened at 8–10 wk for high blood velocity in the ascending thoracic aorta. A male with an aortic blood velocity >7 SD above the mean of previous offspring was identified. It was bred to BALB/c females to test for heritability.

### Genetic mapping, sequencing, and genotyping of point mutation

PCR amplification of individual microsatellite markers using fluorescently tagged primers (IDT, Coralville, IA) was performed on extracted tail DNA from offspring. Labeled products were multiplexed and analyzed on a BaseStation automated sequencer (MJ Research, Waltham, MA) to identify alleles from the mutagenized strain (C57BL/6J). Linkage analysis on BALB/c localized the causative mutation to chromosome 5 (**Figure S1**). Phenotype penetrance was greater on C57BL/6J so mice were then bred to the consomic chromosome substitution strain, C57BL/6J-Chr 5^A/J^/NaJ (www.jax.org, Stock Number 004383) and linkage analysis continued on chromosome 5 from the A/J strain. SNP markers unique to C57BL/6J and A/J strains were used to refine the linkage interval. Genomic sequencing of candidate genes revealed a point mutation in exon 5 of *Klf3* (*Krüppel-like factor 3*) that changed a histidine residue (CAC) at amino acid 275 to arginine (CGC) (KLF3^H275R^). The mouse line has been named *Klf3^m1Jrt^*.

For genotyping the *Klf3* ^H275R^ point mutant mice, we distinguished the mutant *Klf3* DNA from the endogenous wild type DNA using the base pair change from an A to a G, which introduced a novel BstUII restriction enzyme site (**Table S7**).

### Phenotyping

#### Micro-ultrasound imaging and Doppler

The primary screen, high blood velocity in the ascending thoracic aorta, was assessed in isoflurane-anesthetized mice using pulsed Doppler ultrasound (20 MHz transducer; Indus Instruments, Houston, TX) while the rectal temperature was maintained at 37–38°C. Echocardiography was performed in isoflurane-anesthetized adults as a secondary screen (30–40 MHz; Vevo770 or Vevo2100, VisualSonics, Toronto, Canada) using published and/or standard methods [39], [40]. Peak ascending aortic blood velocity was assessed in isoflurane-anesthetized newborn mice one day after birth.

#### Arterial blood pressure

Arterial blood pressure and heart rate were measured in conscious mice using a computerized tail cuff system (MC-4000 Hatteras Instruments, NC; www.cmhd.ca) and/or in isoflurane-anesthetized mice with a catheter-tip pressure transducer (Ultra-Miniature Mikro-Tip; Millar Instruments, TX) inserted into the ascending aorta and left ventricle via the carotid artery.

#### Hematology and blood smears

Whole blood was collected from the saphenous vein into EDTA coated capillary tubes (Drummond Scientific Co., Broomall, PA) and analyzed on a Hemavet 950FS Hematology Analyzer (Drew Scientific, Waterbury, CT). Blood smears were stained with Wright – Giemsa SureStain (Fisher Scientific, Ottawa, Canada).

#### Metabolic rate

Oxygen consumption of males (n = 4 per group, 22–25 wk) was measured over 24 h by indirect calorimetry (Oxymax, Columbus Instruments, Columbus, OH) after 2 h acclimatization to the chamber.

#### Percent body fat

Body composition of anesthetized mice was assessed at 10 wk using Dual energy X-ray absorptiometry (DEXA) (PIXImus, Lunar Corp., Madison, WI) (www.cmhd.ca).

#### Magnetic resonance imaging (MRI), optical projection tomography (OPT), and micro-computed tomography (micro-CT)

Samples for MRI were prepared using methods adapted from [41] and imaged using standard methods [42]. Sample preparation for OPT was essentially as described [43] and images were acquired using standard methods [44], [45]. Micro-CT samples were immersed in iodine [46] to generate soft tissue contrast and imaging was performed as previously published [47], [48].

#### Histopathology

Animals were perfusion fixed with 10% neutral-buffered formalin, tissues were processed, embedded in paraffin, and 5 µm sections stained with hematoxylin and eosin, or Movat's. *LacZ* whole mount staining of embryos and slices of adult tissues were performed using standard methods [49] followed by routine histology.

### Zebrafish morpholino injections

The zebrafish *klf3* (Accession Number NM_131859) sequence was verified and targeted. A morpholino spanning the translation start site of the zebrafish *klf3* gene (sequence: 5′- agcatggctgcttccagtggaattt – 3′) was designed and ordered from Gene Tools (Philomath, Oregon). Following morpholino titration to determine the optimal concentration, 7 ng of morpholino was injected per embryo at the 1-cell stage using standard techniques. The *myl7:EGFP^twu34^* transgenic line was used to image the developing heart.

### Embryonic stem cell lines

Embryonic stem cell lines XS0187 (XS) (MGI:4331780) and CH0516 (CH) (MGI:3872001) from the Sanger Institute Gene Trap Resource were obtained from the *NIH/NCRR-*sponsored Mutant Mouse Regional Resource Center at UC Davis. The location of the gene trap insertion was determined by long range PCR and confirmed by sequencing. 5′ RACE analysis (Sanger Institute Gene Trap Resource) placed the gene trap insertion cassette in *Klf3* in intron 2 for XS and in intron 1 for CH. Primers for long-range PCR were designed at regular intervals spanning the preceding intronic sequence for the 5′ primer and a common sequence at the beginning of the *B-geo fusion* gene was used for the 3′ primer. PCR with each of the long-range primer sets was then undertaken systemically to narrow down the interval of the insertion site as determined by the production of a ∼1–2.5 kbp fragment. The resulting PCR product was purified and sent to The Centre for Applied Genomics (The Hospital for Sick Children, Toronto, Canada) for sequencing. To determine the exact site of insertion, sequencing results were compared to the intronic sequence of the *Klf3* gene and the gene trap vector using the NCBI BLAST programme.

In XS, the gene trap cassette inserted 4105 bp downstream of the 5′ end of intron 2 (4,843 bp in total) (**Figure 8A**). In CH, the gene trap cassette inserted 3578 bp downstream from the start of intron 1 (12,943 bp in total) (**Figure 8A**). Primers were designed for genotyping mutants such that PCR and gel electrophoresis of genomic DNA yielded different sized products for the endogenous WT allele and for the allele with the gene trap insertion (**Table S7**). The mouse lines B6;129-Klf3^Gt(XS0187)Wtsi^/Cmhd (XS) and B6;129-Klf3^Gt(CH0516)Wtsi^/Cmhd (CH) were derived from embryonic stem cells by the Transgenic Core at the Toronto Centre for Phenogenomics.

### Molecular methods

#### Vectors and cloning

The mammalian expression vector pMT3-Klf3 encoding full length murine *Klf3* has been described previously [50]. The bacterial expression vector pGEX-6P-Klf3-F1-3, encoding residues 254–344 of *Klf3* fused to glutathione S-transferase (GST), was kindly provided by Jacqui Matthews and Sandra Wissmueller (School of Molecular Bioscience, University of Sydney, NSW, Australia). The H275R mutation was incorporated into both of these vectors by overlap PCR to generate pMT3-Klf3H275R and pGEX-6P-Klf3-F1-3-H275R respectively.

#### COS transfections, preparation of nuclear extracts and analysis

COS cells were transfected with 1, 2 or 5 µg vector (pMT3, pMT3-Klf3 or pMT3-Klf3H275R) as described previously [9]. After 48 h, nuclear extracts were prepared and subjected to electrophoretic mobility shift assays using a radiolabelled DNA probe containing the mouse *β-major globin* promoter CACCC box as described previously [8]. Western blots were performed as described previously [50] using NuPAGE 10% Bis-Tris gels (Invitrogen, Carlsbad, CA) and antisera specific for KLF3 [8]. Detection was achieved using the Immobilon Western Chemiluminescent HRP Substrate kit (Millipore, Billerica, MA) and X-ray film (Eastman Kodak Company, Rochester, NY).

#### Bacterial expression, GST purification and analysis

Bacteria were transformed with pGEX-6P, pGEX-6P-Klf3-F1-3, and pGEX-6P-Klf3-F1-3-H275R respectively and expressed GST fusion proteins were purified on glutathione beads as described previously [51]. Proteins were analysed by electrophoretic mobility shift assay and by sodium dodecyl sulphate-polyacrylamide gel electrophoresis as above. Polyacrylamide gels were stained with Coomassie blue and subsequently destained by sequential 1 h immersions in solutions of 40% (v/v) methanol, 10% (v/v) acetic acid and 5% (v/v) methanol, 7% (v/v) acetic acid.

#### RNA extraction from embryos and real time PCR

Total RNA from E12.5 whole embryos and dissected livers were extracted using TRIZOL Reagent (Invitrogen), DNase-treated and cleaned up with RNeasy kits (QIAGEN) as described previously [9]. RNA (2.5 µg) was subsequently used as a template for cDNA synthesis using the SuperScript® VILO™cDNA Synthesis Kit (Invitrogen). Quantitative real time PCR (qRT-PCR) was performed as described [9] but using FastStart Universal SYBR Green Master (Rox) (Roche Diagnostics, Indianapolis, IN) or Power SYBR Green PCR Master Mix (Applied Biosystems, Foster City, CA), and the 7500 Fast Real-Time PCR System (Applied Biosystems). Primer sequences are shown in **Table S8**.

#### Nuclear protein extraction from embryos

Nuclear protein extracts were isolated from E12.5 whole embryos, analysed by Western blotting and visualized by chemiluminescence as described previously [50].

### Statistical analysis

Significant differences (P<0.05) were tested using Student's t-test or, if normality failed, a Mann-Whitney Rank Sum Test. Proportions were tested using Chi Square or Fisher's Exact Test. Multiple groups were tested by 2-way or 3-way ANOVA as appropriate, and if significant, then a multiple comparison test was performed. If sex was not a significant factor, then combined data are shown (mean ± SE).

### Microarrays

Total RNA was extracted from whole embryos (E12.5) for the following genotypes: wild type (*n = *4), *Klf3* ^H275R/+^ (*n = *4), *Klf3* ^H275R/H275R^ (*n = *3) and *Klf3* CH homozygous (*n = *4). RNA was prepared and hybridized to Affymetrix GeneChIP 2.0 ST arrays (Affymetrix, Santa Clara, CA) by the Ramaciotti Centre (University of New South Wales, Australia) as previously described [14]. Data were normalized using the robust multiarray average (RMA) algorithm and analyzed using Partek Genomic Suite version 6.6 (Partek Inc., St Louis, MO). Genes that showed greater than 1.5-fold deregulation of expression in *Klf3* ^H275R/H275R^ samples relative to wild type were used to construct the heat map (**Figure S9**). Microarray data were deposited in the Gene Expression Omnibus (http://www.ncbi.nlm.nih.gov/projects/geo) under accession number GSE43908.

## Supporting Information

Figure S1 Mapping mutation in ENU mutant line. (A) Genome scan of mice with high aortic blood velocity trait. Microsatellite markers polymorphic between parental strains (H) localized mutation between markers D5Mit346 and D5Mit201. (B) LOD score of trait on chromosome 5. Vertical dashed lines show fine mapped interval, which contained 35 genes. Vertical solid line marked with an arrow shows the location of *Klf3* in which the potentially causative point mutation was found by sequencing.
(TIF)

Figure S2 Histology of the pulmonary valve in adult *Klf3* mutants. Pulmonary valve histology at low power (A,C) for wild type (WT) mice (left) and (A) for 2 different *Klf3* ^H275R^/+ mutants (middle and right) and (C) for homozygous XS (middle) and CH mutants (right). (B,D) Higher power images of the pulmonary valves located in the boxed regions in (A,C). Pulmonary valve abnormalities were not detected in adult *Klf3* ^H275R^/+ mutants by gross or histological examination. Pulmonary valve leaflets were often abnormally thickened in XS and CH mutants (arrows).
(TIF)

Figure S3 Enlarged heart of an adult *Klf3* ^H275R^ heterozygote that was found moribund. Heart image at 47 wk of a moribund *Klf3* ^H275R^ heterozygote (right) in comparison to a littermate control (left). Images show the dramatic cardiac enlargement typical of *Klf3* ^H275R^ heterozygotes that became acutely ill and were found moribund, likely due to high output heart failure. Images show 3D micro-CT surface renderings of isolated hearts.
(TIF)

Figure S4 Light and electron microscopy of the adult ventricular myocardium. In general, the structure of the mutant myocardium appeared normal when examined (A) by light microscopy, and (B) by electron microscopy. However, focal regions of contraction band necrosis (arrows) were sometimes observed in the *Klf3^H275R^*/+ myocardium (right) whereas this was rare in the wild type myocardium (left).
(TIF)

Figure S5 Hematological parameters in adult *Klf3* mutants. Blood of *Klf3* ^H275R^ and of XS and CH gene trap lines was sampled at 9–19 wk. RBC = red blood cell counts; MCV = red blood cell volume; RDW = red blood cell distribution width; WBC = white blood cell count; WT = wild-type; Het = heterozygotes; Homo = homozygotes. No homozygote *Klf3^H275R^* mice survived to adulthood (N/A). N is shown in bar. * P<0.05 ** P<0.01 *** P<0.001 vs. WT, NS = not significant. Mean ± SE.
(TIF)

Figure S6 KLF3^H275R^ protein does not interfere with the ability of WT KLF3 to bind to DNA. No reduction in binding of WT KLF3 to KLF3's canonical CACCC binding region of the β-globin gene promoter was observed when (A) the recombinant WT protein was combined with recombinant bacterial GST-*Klf3* ^H275R^ zinc finger 1–3 protein in ratios of 1∶1, 1∶2, and 1∶4 or (B) when full length WT protein was combined with KLF3^H275R^ protein expressed in COS cells at a 1∶1 ratio. These results show that KLF3^H275R^ protein does not interfere with the ability of WT KLF3 to bind to DNA.
(TIF)

Figure S7 Increased mRNA expression of *Lgals3* in *Klf3* ^H275R^ mutant embryos. *Lgals3* mRNA was significantly increased in (A) homozygous (Homo) *Klf3* ^H275R^ embryos at E12.5 relative to wild type littermate controls (WT). (B) *Lgals3* was significantly increased in livers from homozygous (Homo) and heterozygous (Het) *Klf3* ^H275R^ embryos at E12.5. These results show that the point mutation impairs the normal repressive function of *Klf3* at this target gene *in vivo*. qRT-PCR expression was normalized to 18S (reference gene) and to the WT group mean ( = 1). Results for individual embryos are shown. Horizontal lines shows group means. * P<0.05 relative to WT.
(TIF)

Figure S8 LacZ staining in the adult heart and aorta showing *Klf3* gene expression. (A) LacZ-staining (blue) in homozygous XS mice shows *Klf3* gene expression in the atrial myocardium, left atrioventricular valve, left ventricular myocardium, and aorta. Lower magnification images are shown on left (20×) and higher magnification images on right (100×). (B) Images (100×) at similar anatomic locations in wild type (WT) mice showing no detectable Lac-Z staining (negative control).
(TIF)

Figure S9 Heat map showing relative microarray gene expression of *Klf3* ^H275R^ and CH mutants versus wild type. Genes with a greater than 1.5-fold difference in expression in *Klf3* ^H275R/H275R^ embryos relative to wild type are shown. These differences were not statistically significant at a false discovery rate of 0.1. *Lilra6* and *Hsd3b6* mRNA was measured by qRT-PCR. RNA was prepared from whole embryos at E12.5 and was analyzed by Affymetrix microarrays for n = 4 wild type, n = 4 *Klf3* ^H275R/+^, n = 3 *Klf3* ^H275R/H275R^ and n = 4 *Klf3* CH homozygous gene trap embryos.
(TIF)

Figure S10 Activator and repressor functions of *Klf3* ^H275R^ in mutant embryos. (A) Activation of *Hsd3b6* was significantly diminished in heterozygous (Het) and homozygous (Homo) *Klf3* ^H275R^ embryos at E12.5 relative to wild type littermate controls (WT). (B) A trend towards a similar diminishment in *Hsd3b6* expression in homozygous CH gene trap mutants was not statistically significant (whereas *Hsd3b6* expression was significantly reduced when assessed by microarray analysis (**Table S6A**)). (C) *Lilra6* mRNA expression was significantly augmented in heterozygous (Het) and homozygous (Homo) *Klf3* ^H275R^ embryos at E12.5 relative to wild type littermate controls (WT). (D) *Lilra6* mRNA expression in homozygous CH gene trap mutants was not significantly altered, nor was expression in homozygous CH mutants significantly altered when assessed by microarray analysis. Results suggest that the point mutation diminished the activator function of KLF3 at *Hsd3b6*, and that it generated a novel activating function on *Lilra6* mRNA expression in embryos *in vivo*. qRT-PCR expression was normalized to 18S (reference gene) and the WT group mean ( = 1). Bars show the mean ± SE. The number of embryos is shown at the bottom of each bar. * P<0.05 relative to WT (by Kruskal-Wallis ANOVA on Ranks).
(TIF)

Movie S1 Motion of the inter-ventricular septum during the cardiac cycle in adult wild type mice. Short axis view of the inter-ventricular septum, and right and left ventricles of a wild type adult mouse (58 wk) obtained using micro-ultrasound. The septum is near the middle of the image with the right ventricle on the left side, and the left ventricle on the right side.
(MOV)

Movie S2 Abnormal motion of the inter-ventricular septum during the cardiac cycle in adult CH homozygous mice. Short axis view of the inter-ventricular septum, and right and left ventricles of a homozygous Klf3 gene trap mutant of the CH line (age 58 wk) obtained using micro-ultrasound. The septum is near the middle of the image with the visibly enlarged right ventricle on the left side, and the left ventricle on the right side. At the end of systole, the inter-ventricular septum suddenly deforms and bulges into the left ventricle. This is likely caused by right ventricular systole continuing after the end of left ventricular systole due to the marked enlargement in right ventricular size in the CH mutant. Similar anatomic and ultrasound defects were also observed in some adult homozygotes of the XS line.
(MOV)

Table S1 List of 35 genes in mapping interval on Chromosome 5.
(DOCX)

Table S2 Stage of perinatal or postnatal lethality in *Klf3* ^H272R^ mutants.
(DOCX)

Table S3 Body and organ weights of *Klf3* ^H275R^ heterozygous mice at 18–25 wk.
(DOCX)

Table S4 Lethality at weaning of XS and CH gene trap mutants.
(DOCX)

Table S5 Body and organ weights of XS and CH homozygous adult mice at 18–82 wk.
(DOCX)

Table S6 Differential gene expression of embryos at E12.5 by microarray with False Discovery Rate (FDR) <0.1. A. WT (n = 4) versus CH homozygotes (n = 4). B. *Klf3* ^H275R^ homozygotes (n = 3) versus CH homozygotes (n = 4). C. *Klf3* ^H275R^ heterozygotes (n = 4) versus CH homozygotes (n = 4).
(DOCX)

Table S7 PCR primers for genomic DNA used to genotype mouse lines.
(DOCX)

Table S8 qRT-PCR primer sequences for mRNA.
(DOCX)
