# Supplementary figures and images for: ENU-induced Mutation in the DNA-binding Domain of KLF3 Reveals Important Roles for KLF3 in Cardiovascular Development and Function in Mice
Source: PLoS Genet. 2013 Jul 11;9(7):e1003612. doi: 10.1371/journal.pgen.1003612 (PMC3708807; doi:10.1371/journal.pgen.1003612)

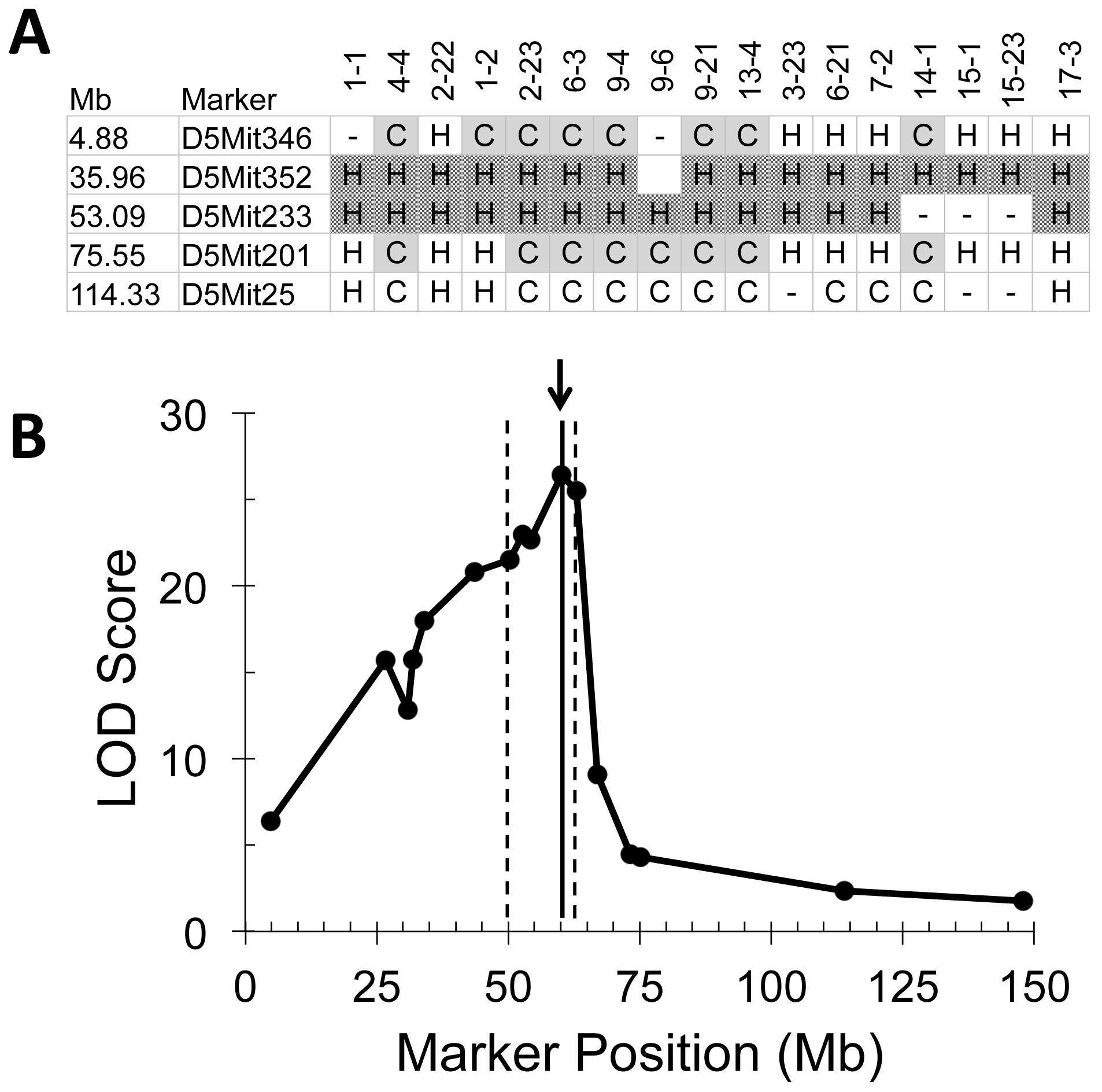

Supplement: Figure S1 — Mapping mutation in ENU mutant line. (A) Genome scan of mice with high aortic blood velocity trait. Microsatellite markers polymorphic between parental strains (H) localized mutation between markers D5Mit346 and D5Mit201. (B) LOD score of trait on chromosome 5. Vertical dashed lines show fine mapped interval, which contained 35 genes. Vertical solid line marked with an arrow shows the location of Klf3 in which the potentially causative point mutation was found by sequencing. (TIF) [file pgen.1003612.s001.tif]

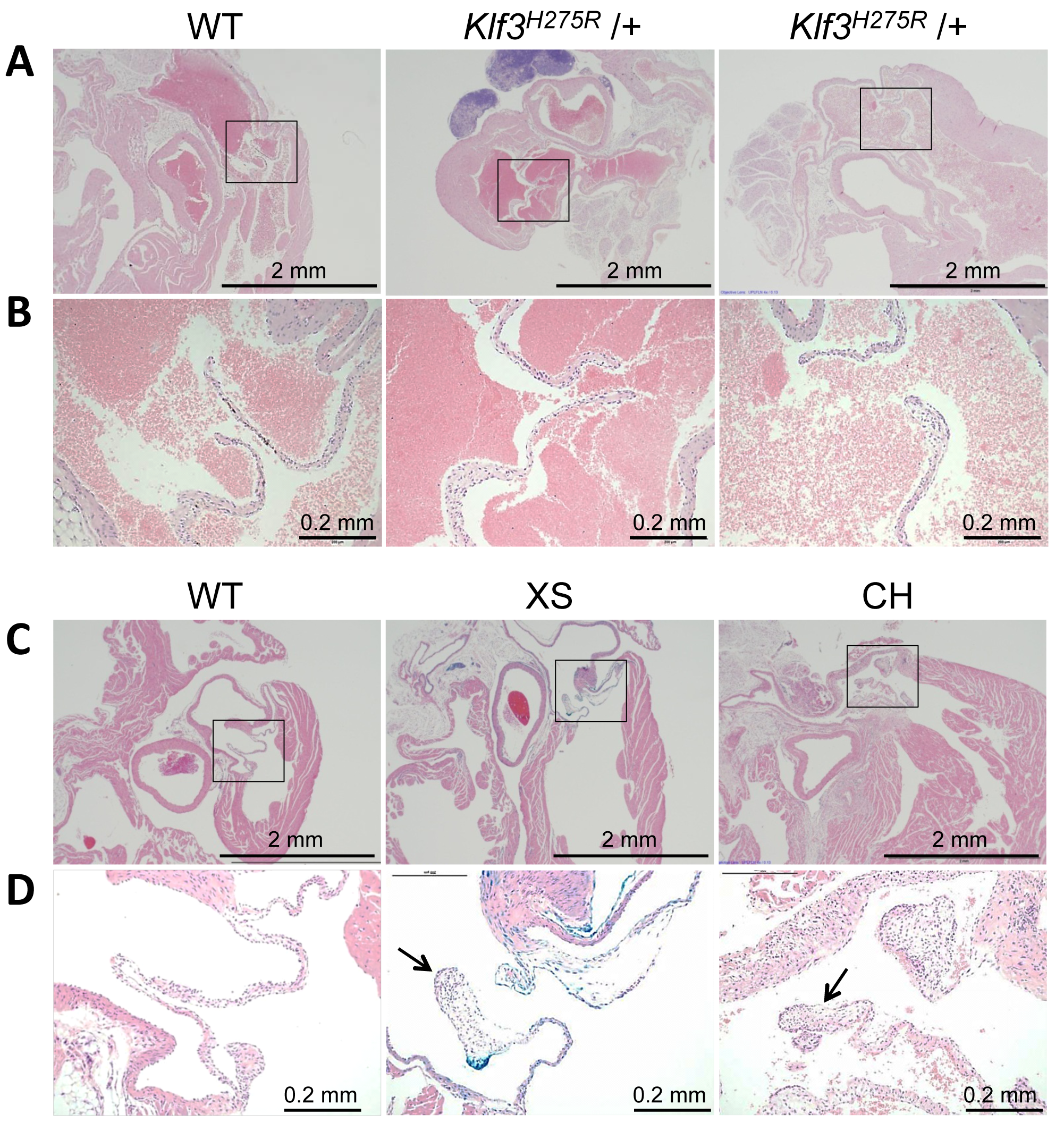

Supplement: Figure S2 — Histology of the pulmonary valve in adult Klf3 mutants. Pulmonary valve histology at low power (A,C) for wild type (WT) mice (left) and (A) for 2 different Klf3 H275R/+ mutants (middle and right) and (C) for homozygous XS (middle) and CH mutants (right). (B,D) Higher power images of the pulmonary valves located in the boxed regions in (A,C). Pulmonary valve abnormalities were not detected in adult Klf3 H275R/+ mutants by gross or histological examination. Pulmonary valve leaflets were often abnormally thickened in XS and CH mutants (arrows). (TIF) [file pgen.1003612.s002.tif]

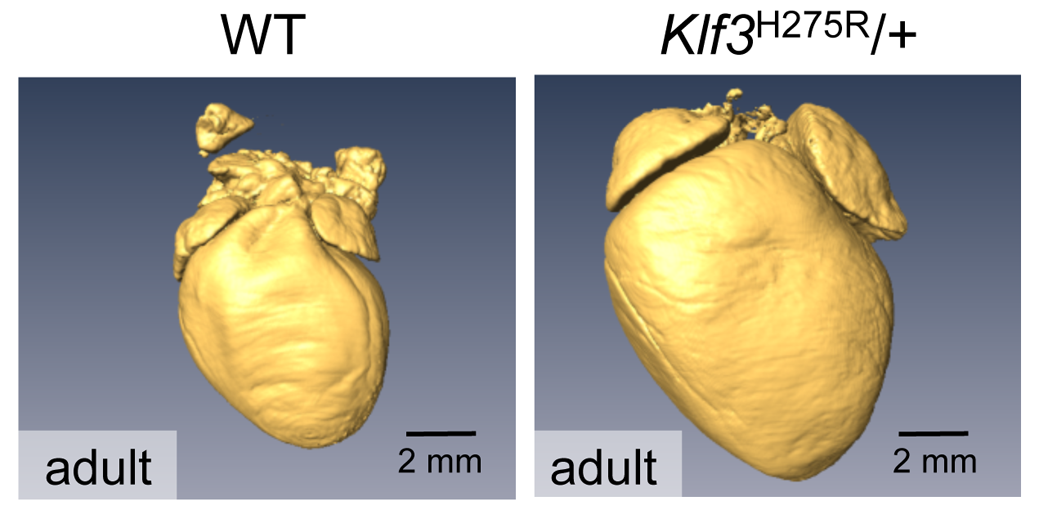

Supplement: Figure S3 — Enlarged heart of an adult Klf3 H275R heterozygote that was found moribund. Heart image at 47 wk of a moribund Klf3 H275R heterozygote (right) in comparison to a littermate control (left). Images show the dramatic cardiac enlargement typical of Klf3 H275R heterozygotes that became acutely ill and were found moribund, likely due to high output heart failure. Images show 3D micro-CT surface renderings of isolated hearts. (TIF) [file pgen.1003612.s003.tif]

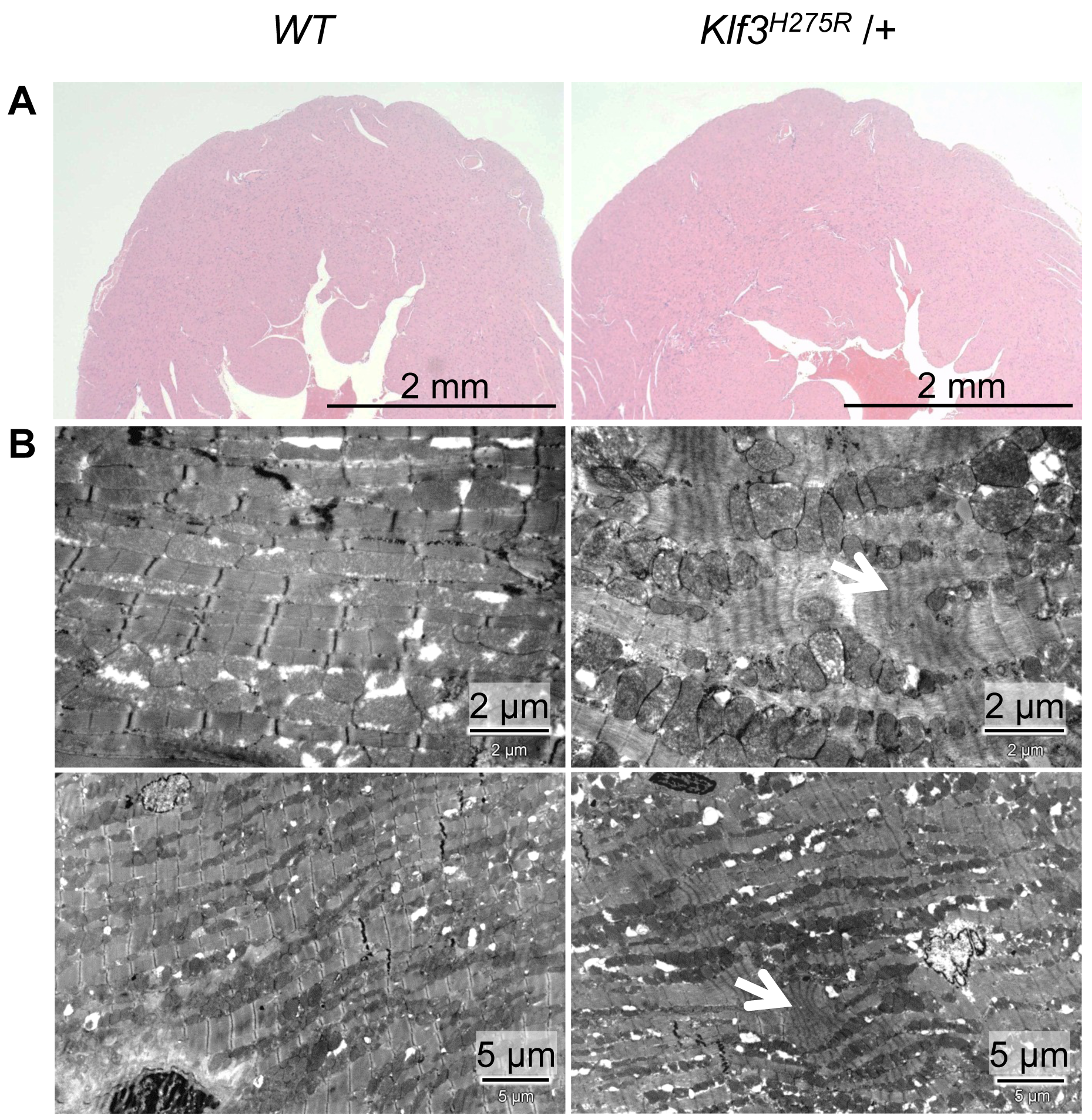

Supplement: Figure S4 — Light and electron microscopy of the adult ventricular myocardium. In general, the structure of the mutant myocardium appeared normal when examined (A) by light microscopy, and (B) by electron microscopy. However, focal regions of contraction band necrosis (arrows) were sometimes observed in the Klf3H275R/+ myocardium (right) whereas this was rare in the wild type myocardium (left). (TIF) [file pgen.1003612.s004.tif]

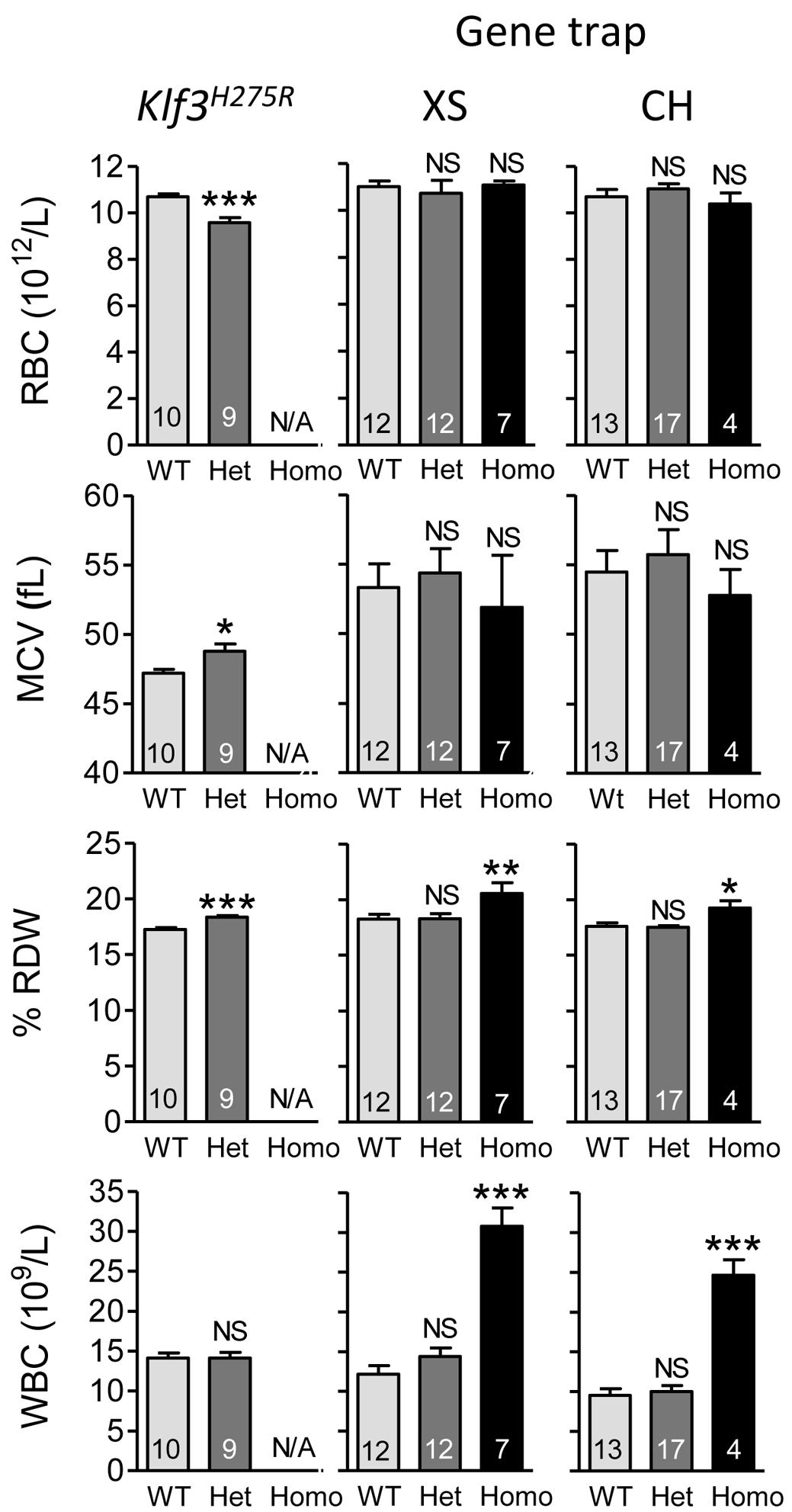

Supplement: Figure S5 — Hematological parameters in adult Klf3 mutants. Blood of Klf3 H275R and of XS and CH gene trap lines was sampled at 9–19 wk. RBC = red blood cell counts; MCV = red blood cell volume; RDW = red blood cell distribution width; WBC = white blood cell count; WT = wild-type; Het = heterozygotes; Homo = homozygotes. No homozygote Klf3H275R mice survived to adulthood (N/A). N is shown in bar. * P<0.05 ** P<0.01 *** P<0.001 vs. WT, NS = not significant. Mean ± SE. (TIF) [file pgen.1003612.s005.tif]

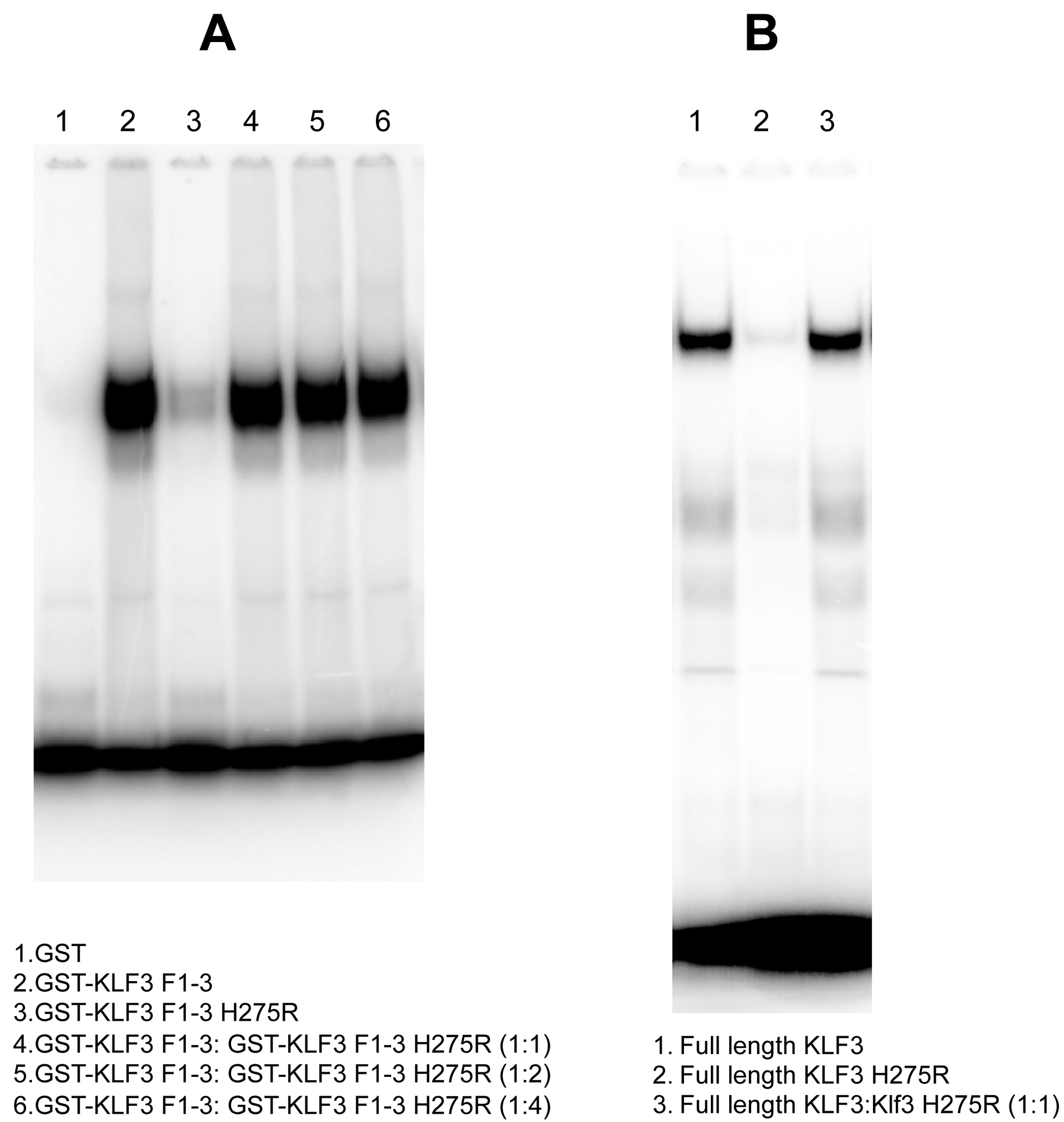

Supplement: Figure S6 — KLF3H275R protein does not interfere with the ability of WT KLF3 to bind to DNA. No reduction in binding of WT KLF3 to KLF3's canonical CACCC binding region of the β-globin gene promoter was observed when (A) the recombinant WT protein was combined with recombinant bacterial GST-Klf3 H275R zinc finger 1–3 protein in ratios of 1∶1, 1∶2, and 1∶4 or (B) when full length WT protein was combined with KLF3H275R protein expressed in COS cells at a 1∶1 ratio. These results show that KLF3H275R protein does not interfere with the ability of WT KLF3 to bind to DNA. (TIF) [file pgen.1003612.s006.tif]

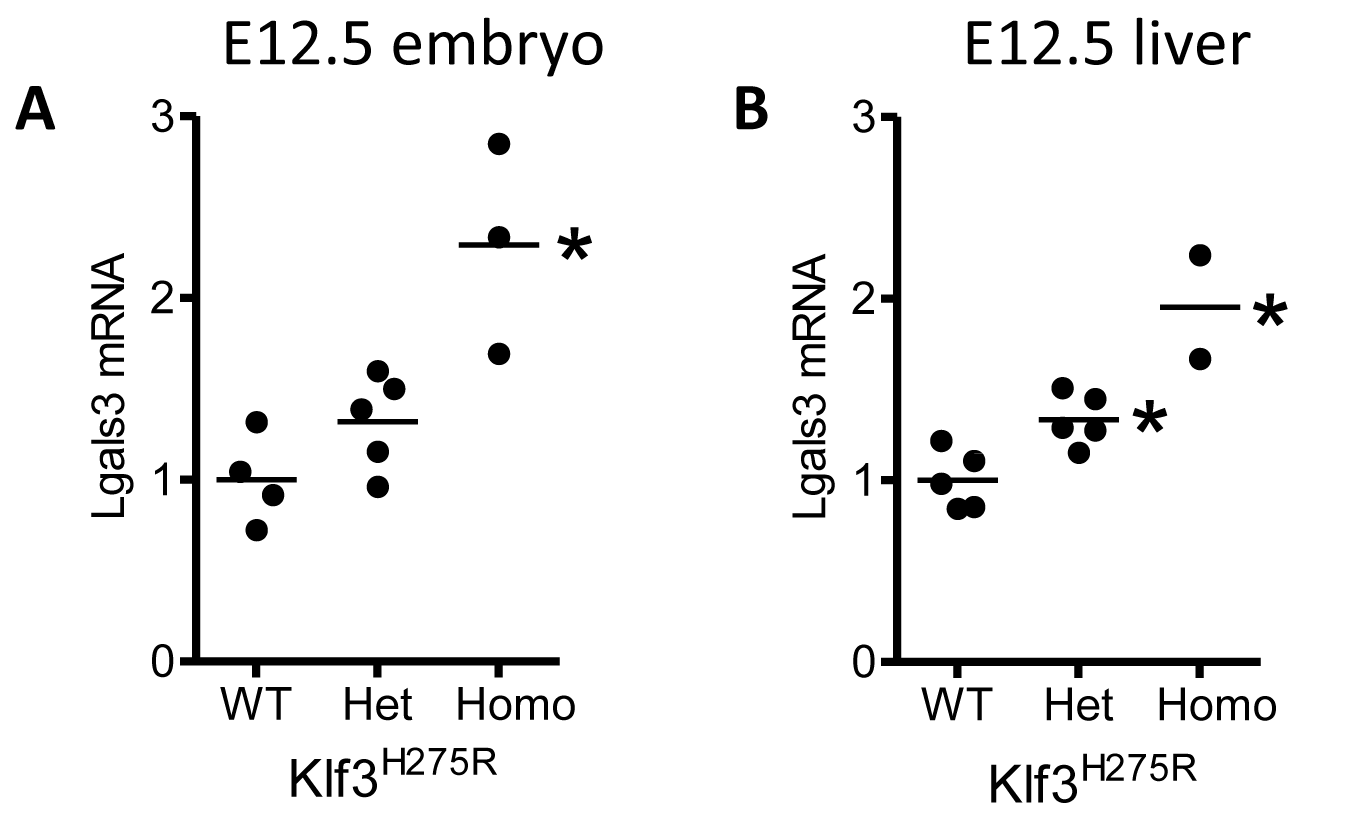

Supplement: Figure S7 — Increased mRNA expression of Lgals3 in Klf3 H275R mutant embryos. Lgals3 mRNA was significantly increased in (A) homozygous (Homo) Klf3 H275R embryos at E12.5 relative to wild type littermate controls (WT). (B) Lgals3 was significantly increased in livers from homozygous (Homo) and heterozygous (Het) Klf3 H275R embryos at E12.5. These results show that the point mutation impairs the normal repressive function of Klf3 at this target gene in vivo. qRT-PCR expression was normalized to 18S (reference gene) and to the WT group mean ( = 1). Results for individual embryos are shown. Horizontal lines shows group means. * P<0.05 relative to WT. (TIF) [file pgen.1003612.s007.tif]

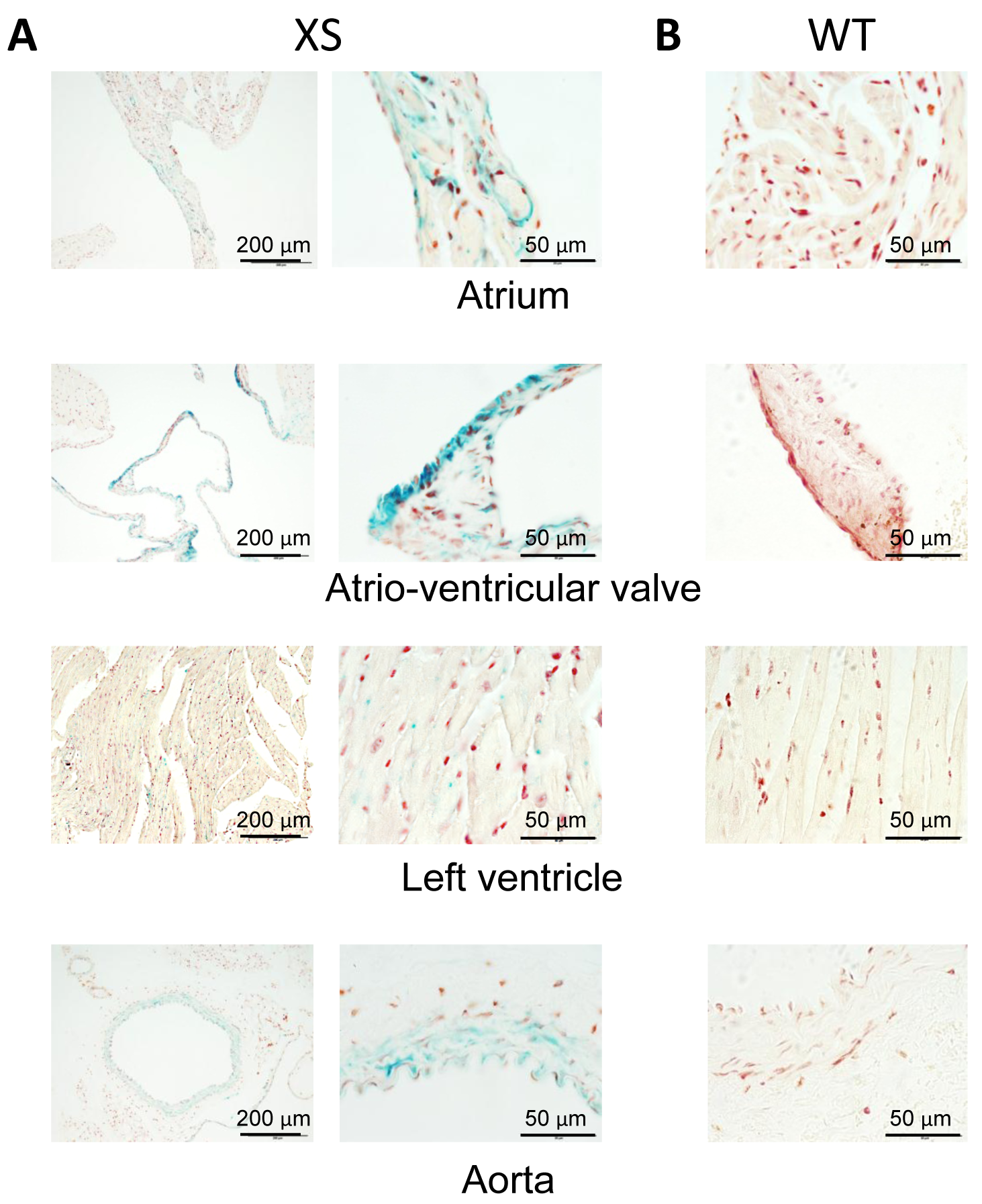

Supplement: Figure S8 — LacZ staining in the adult heart and aorta showing Klf3 gene expression. (A) LacZ-staining (blue) in homozygous XS mice shows Klf3 gene expression in the atrial myocardium, left atrioventricular valve, left ventricular myocardium, and aorta. Lower magnification images are shown on left (20×) and higher magnification images on right (100×). (B) Images (100×) at similar anatomic locations in wild type (WT) mice showing no detectable Lac-Z staining (negative control). (TIF) [file pgen.1003612.s008.tif]

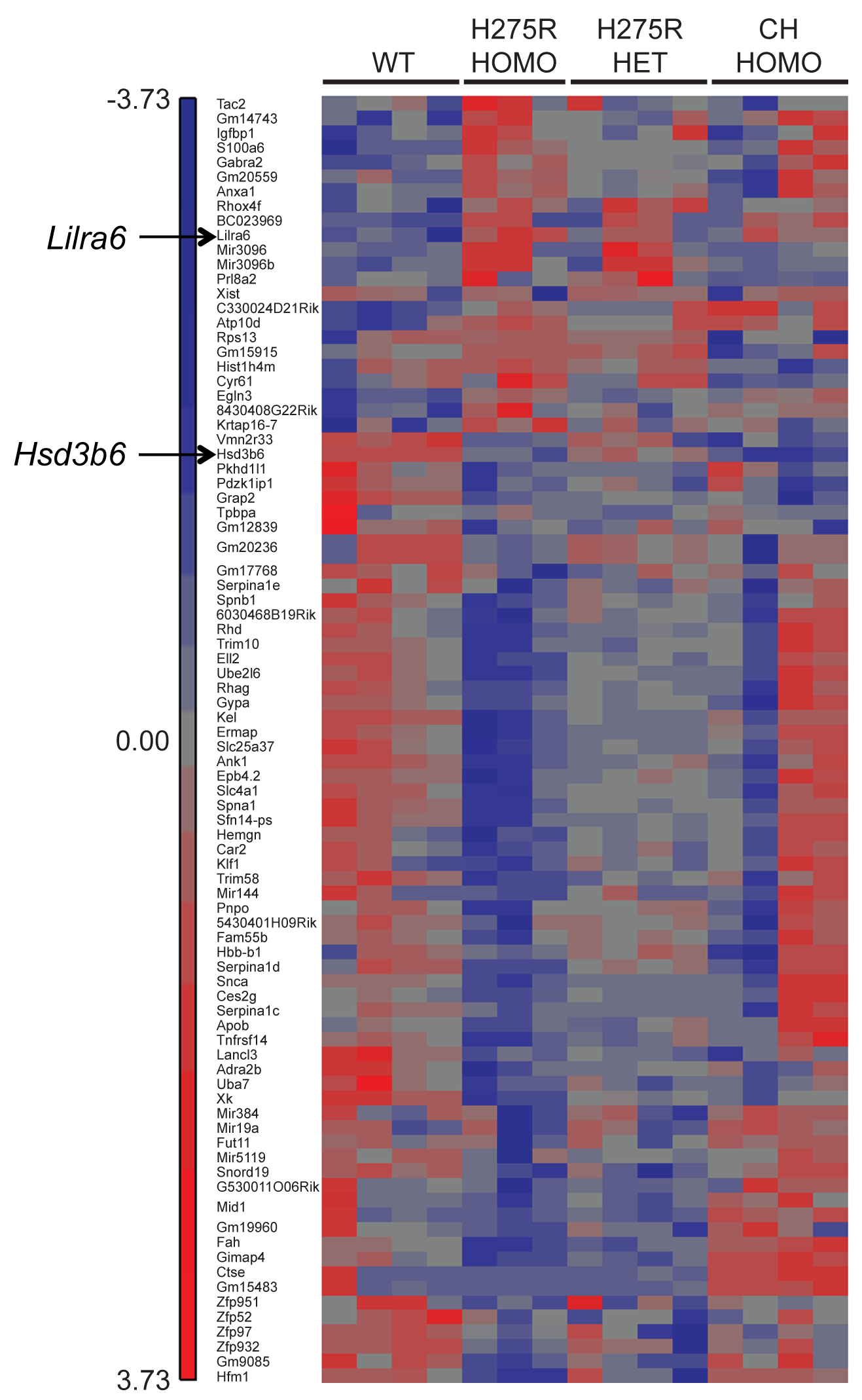

Supplement: Figure S9 — Heat map showing relative microarray gene expression of Klf3 H275R and CH mutants versus wild type. Genes with a greater than 1.5-fold difference in expression in Klf3 H275R/H275R embryos relative to wild type are shown. These differences were not statistically significant at a false discovery rate of 0.1. Lilra6 and Hsd3b6 mRNA was measured by qRT-PCR. RNA was prepared from whole embryos at E12.5 and was analyzed by Affymetrix microarrays for n = 4 wild type, n = 4 Klf3 H275R/+, n = 3 Klf3 H275R/H275R and n = 4 Klf3 CH homozygous gene trap embryos. (TIF) [file pgen.1003612.s009.tif]

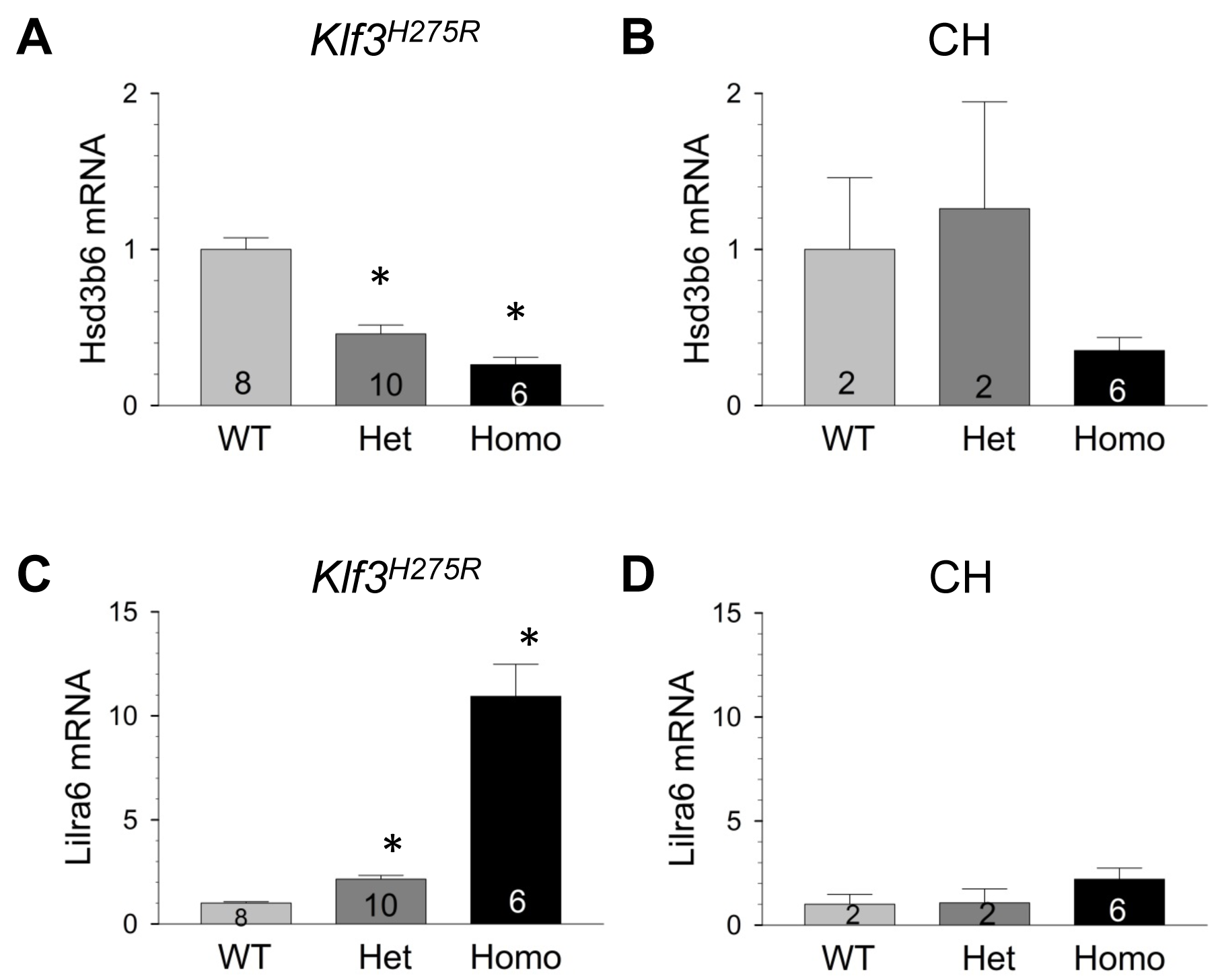

Supplement: Figure S10 — Activator and repressor functions of Klf3 H275R in mutant embryos. (A) Activation of Hsd3b6 was significantly diminished in heterozygous (Het) and homozygous (Homo) Klf3 H275R embryos at E12.5 relative to wild type littermate controls (WT). (B) A trend towards a similar diminishment in Hsd3b6 expression in homozygous CH gene trap mutants was not statistically significant (whereas Hsd3b6 expression was significantly reduced when assessed by microarray analysis (Table S6A)). (C) Lilra6 mRNA expression was significantly augmented in heterozygous (Het) and homozygous (Homo) Klf3 H275R embryos at E12.5 relative to wild type littermate controls (WT). (D) Lilra6 mRNA expression in homozygous CH gene trap mutants was not significantly altered, nor was expression in homozygous CH mutants significantly altered when assessed by microarray analysis. Results suggest that the point mutation diminished the activator function of KLF3 at Hsd3b6, and that it generated a novel activating function on Lilra6 mRNA expression in embryos in vivo. qRT-PCR expression was normalized to 18S (reference gene) and the WT group mean ( = 1). Bars show the mean ± SE. The number of embryos is shown at the bottom of each bar. * P<0.05 relative to WT (by Kruskal-Wallis ANOVA on Ranks). (TIF) [file pgen.1003612.s010.tif]
