# Supplementary material for: ENU-induced Mutation in the DNA-binding Domain of KLF3 Reveals Important Roles for KLF3 in Cardiovascular Development and Function in Mice
Source: PLoS Genet. 2013 Jul 11;9(7):e1003612. doi: 10.1371/journal.pgen.1003612 (PMC3708807; doi:10.1371/journal.pgen.1003612)
Supplement: Table S1 — List of 35 genes in mapping interval on Chromosome 5. (DOCX) [file pgen.1003612.s013.docx]

**Table S1. List of 35 genes in mapping interval on Chromosome 5.**

| **Start Position (bp)** | **Strand** | **Ensembl Gene ID** | **External Gene ID** |
| --- | --- | --- | --- |
| 53809626 | + | ENSMUSG00000039178 | Tbc1d19 |
| 53998417 | + | ENSMUSG00000039156 | **Stim2*** |
| 57718021 | + | ENSMUSG00000029108 | **Pcdh7*** |
| 59053599 | + | ENSMUSG00000094696 | AC122012.1 |
| 61808843 | + | ENSMUSG00000089992 | G6pd2 |
| 62602445 | - | ENSMUSG00000037999 | Arap2 |
| 62813823 | + | ENSMUSG00000090326 | Gm17384 |
| 63649103 | + | ENSMUSG00000090061 | 3110047P20Rik |
| 63649950 | - | ENSMUSG00000054714 | Gm9954 |
| 63812495 | + | ENSMUSG00000060512 | 0610040J01Rik |
| 63908896 | - | ENSMUSG00000047881 | Rell1 |
| 64092936 | + | ENSMUSG00000029171 | Pgm1 |
| 64159451 | + | ENSMUSG00000029174 | Tbc1d1 |
| 64803523 | + | ENSMUSG00000029178 | **Klf3*** |
| 64924811 | - | ENSMUSG00000044827 | Tlr1 |
| 64953106 | - | ENSMUSG00000051498 | Tlr6 |
| 64970071 | + | ENSMUSG00000029185 | Fam114a1 |
| 65063208 | - | ENSMUSG00000037913 | Tmem156 |
| 65107568 | + | ENSMUSG00000054920 | Klhl5 |
| 65199696 | + | ENSMUSG00000037890 | Wdr19 |
| 65261852 | - | ENSMUSG00000029191 | Rfc1 |
| 65348386 | + | ENSMUSG00000029195 | **Klb*** |
| 65388364 | - | ENSMUSG00000047215 | Rpl9 |
| 65391497 | + | ENSMUSG00000029199 | Lias |
| 65413202 | - | ENSMUSG00000029201 | **Ugdh*** |
| 65446844 | - | ENSMUSG00000037822 | 1110003E01Rik |
| 65537233 | + | ENSMUSG00000028203 | Ube2k |
| 65615260 | - | ENSMUSG00000029202 | Pds5a |
| 65763521 | + | ENSMUSG00000037795 | N4bp2 |
| 65863569 | + | ENSMUSG00000029204 | **Rhoh*** |
| 65934921 | + | ENSMUSG00000029205 | **Chrna9*** |
| 65979358 | - | ENSMUSG00000054598 | 9130230L23Rik |
| 66016549 | - | ENSMUSG00000070780 | Rbm47 |
| 66259912 | + | ENSMUSG00000029206 | Nsun7 |
| 66298861 | - | ENSMUSG00000029207 | Apbb2 |

***Genes in bold font were sequenced.**
