# Supplementary material for: ENU-induced Mutation in the DNA-binding Domain of KLF3 Reveals Important Roles for KLF3 in Cardiovascular Development and Function in Mice
Source: PLoS Genet. 2013 Jul 11;9(7):e1003612. doi: 10.1371/journal.pgen.1003612 (PMC3708807; doi:10.1371/journal.pgen.1003612)
Supplement: Table S2 — Stage of perinatal or postnatal lethality in Klf3 H272R mutants. (DOCX) [file pgen.1003612.s014.docx]

Table S2. Stage of perinatal or postnatal lethality in *Klf3*^H272R^ mutants.

| **Genotype** | **E12.5** | | | **E14.5-E16.5** | | | **Birth** | | | **Neonate** | | **Weaning** | |
| --- | --- | --- | --- | --- | --- | --- | --- | --- | --- | --- | --- | --- | --- |
|  | **If 1:2:1** | **#** | **# alive** | **If 1:2:1** | **#** | **# alive** | **If 1:1** | **#** | **# alive** | **If 1:1** | **# alive** | **If 1:1** | **# alive** |
| **Wild Type** | 14 | 14 | 14 | 16 | 16 | 16 | 10 | 10 | 9 | 23 | 23 | 189 | 189 |
| **Heterozygote** | 28 | 31 | 27 | 32 | 36 | 36 | 10 | 12 | 7 | 23 | 9 | 189 | 103 |
| **Homozygote** | 14 | 9 | 9 | 16 | 12 | 3 |  |  |  |  |  |  |  |
| **P value*** |  |  | NS |  |  | <0.003 |  |  | NS |  | 0.01 |  | 0.0001 |

* P value for genotype proportion of live offspring differing from expected Mendelian ratio of 1:2:1 for embryos from heterozygous intercross mating, and 1:1 for postnatal offspring from heterozygous x C57Bl6/J mating.
