# Supplementary material for: ENU-induced Mutation in the DNA-binding Domain of KLF3 Reveals Important Roles for KLF3 in Cardiovascular Development and Function in Mice
Source: PLoS Genet. 2013 Jul 11;9(7):e1003612. doi: 10.1371/journal.pgen.1003612 (PMC3708807; doi:10.1371/journal.pgen.1003612)
Supplement: Table S3 — Body and organ weights of Klf3 H275R heterozygous mice at 18–25 wk. (DOCX) [file pgen.1003612.s015.docx]

**Table S3**. Body and organ weights of *Klf3*^H275R^ heterozygous mice at 18-25 wk.

|  | **WT** | ***Klf3*^H275R/+^** | **% change** | **P value** |
| --- | --- | --- | --- | --- |
| body weight (g) | 28 ± 2 | 25 ± 2 | - 12 | NS |
| fat (mg/g)† | 24 ± 4 | 14 ± 2 | - 43 | P<0.05 |
| heart (mg/g) | 6.5 ± 0.5 | 9.3 ± 0.4 | + 43 | P<0.001 |
| spleen (mg/g) | 2.9 ± 0.2 | 4.3 ± 0.3 | + 49 | P<0.001 |
| kidney (mg/g) | 11.8 ± 0.3 | 13.5 ± 0.5 | + 15 | P<0.01 |
| brain (mg/g) | 17 ± 2 | 20 ± 1 | + 15 | NS |
| lung (mg/g) | 6.3 ± 0.2 | 6.6 ± 0.2 | + 4 | NS |
| liver (mg/g) | 42 ± 1 | 44 ± 2 | + 5 | NS |
| age (wk) | 21 ± 0.6 | 21 ± 0.6 | 0 | NS |

Mean ± SEM with N=10 per group; NS, not significant by Student’s t-test; organ weight in mg is expressed per g body weight; †, ventral intraperitoneal fat pad from the lower abdomen (includes uterine fat in females and testicular fat in males)
