# Supplementary material for: ENU-induced Mutation in the DNA-binding Domain of KLF3 Reveals Important Roles for KLF3 in Cardiovascular Development and Function in Mice
Source: PLoS Genet. 2013 Jul 11;9(7):e1003612. doi: 10.1371/journal.pgen.1003612 (PMC3708807; doi:10.1371/journal.pgen.1003612)
Supplement: Table S4 — Lethality at weaning of XS and CH gene trap mutants. (DOCX) [file pgen.1003612.s016.docx]

Table S4. Lethality at weaning of XS and CH gene trap mutants.

| **Genotype** | **XS** | | **CH** | |
| --- | --- | --- | --- | --- |
|  | If 1:2:1 | # alive | If 1:2:1 | # alive |
| **Wild Type** | 21 | 21 | 24 | 24 |
| **Heterozygote** | 42 | 58 | 48 | 51 |
| **Homozygote** | 21 | 7 | 24 | 6 |
| **P value*** |  | 0.00005 |  | 0.0012 |

* P value for genotype proportion of live offspring from heterozygous intercross mating differing from expected Mendelian ratio of 1:2:1.
