# Supplementary material for: ENU-induced Mutation in the DNA-binding Domain of KLF3 Reveals Important Roles for KLF3 in Cardiovascular Development and Function in Mice
Source: PLoS Genet. 2013 Jul 11;9(7):e1003612. doi: 10.1371/journal.pgen.1003612 (PMC3708807; doi:10.1371/journal.pgen.1003612)
Supplement: Table S5 — Body and organ weights of XS and CH homozygous adult mice at 18–82 wk. (DOCX) [file pgen.1003612.s017.docx]

**Table S5. Body and organ weights of XS and CH homozygous adult mice at 18-82 wk.**

|  | WT* | XS and CH* | % change | P value |
| --- | --- | --- | --- | --- |
| body weight (g) | 41 ± 4 (11) | 30 ± 2 (14) | - 27 | P<0.01 |
| fat (mg/g)† | 53 ± 11 (11) | 18 ± 4 (14) | - 67 | P<0.01 |
| heart (mg/g) | 4.7 ± 0.3 (11) | 7.2 ± 0.6 (14) | + 54 | P<0.01 |
| spleen (mg/g) | 3.1 ± 0.8 (11) | 5.4 ± 0.7 (14) | + 73 | P<0.05 |
| kidney (mg/g) | 10.1 ± 0.6 (9) | 15.7 ± 1.6 (11) | + 55 | P<0.01 |
| brain (mg/g) | 13 ± 1 (9) | 17 ± 1 (10) | + 36 | P<0.01 |
| lung (mg/g) | 4.3 ± 0.3 (9) | 7.1 ± 0.9 (11) | + 66 | P<0.05 |
| liver (mg/g) | 40 ± 1 (9) | 46 ± 2 (11) | + 15 | P<0.05 |
| age (wk) | 37 ± 8 (11) | 36 ± 5 (14) | - 2 | NS |

Mean ± SE (n); * pooled data for XS and CH homozygotes and WT cage-mate controls.

Organ weight in mg is expressed per g body weight; †, ventral intraperitoneal fat pad from the lower abdomen (includes uterine fat in females and testicular fat in males); NS, not significant by Student’s t-test.
