# Supplementary material for: ENU-induced Mutation in the DNA-binding Domain of KLF3 Reveals Important Roles for KLF3 in Cardiovascular Development and Function in Mice
Source: PLoS Genet. 2013 Jul 11;9(7):e1003612. doi: 10.1371/journal.pgen.1003612 (PMC3708807; doi:10.1371/journal.pgen.1003612)
Supplement: Table S6 — Differential gene expression of embryos at E12.5 by microarray with False Discovery Rate (FDR) <0.1. A. WT (n = 4) versus CH homozygotes (n = 4). B. Klf3 H275R homozygotes (n = 3) versus CH homozygotes (n = 4). C. Klf3 H275R heterozygotes (n = 4) versus CH homozygotes (n = 4). (DOCX) [file pgen.1003612.s018.docx]

**Table S6. Differential gene expression of embryos at E12.5 by microarray with FDR < 0.1.**

A. WT (n=4) versus CH homozygotes (n=4)

|  | **Gene Symbol** | **RefSeq** | **FDR** | **WT**  **CH Homo** | **CH Homo**  **WT** |
| --- | --- | --- | --- | --- | --- |
| 1 | Klf3 | NM_008453 | 0.000 | 15.14 | 0.07 |
| 2 | Hsd3b6 | NM_013821 | 0.003 | 4.37 | 0.23 |
| 3 | Myh9 | NM_022410 | 0.020 | 1.61 | 0.62 |
| 4 | Ncan | NM_007789 | 0.095 | 1.38 | 0.72 |
| 5 | C630020P19Rik | NR_040354 | 0.095 | 1.29 | 0.77 |
| 6 | Slc9a3r2 | NM_023055 | 0.073 | 1.24 | 0.81 |
| 7 | F2rl2 | ENSMUST00000022182 | 0.074 | 1.18 | 0.85 |
| 8 | Btrc | NM_001037758 | 0.073 | 1.16 | 0.86 |
| 9 | Dguok | NM_013764 | 0.055 | 1.14 | 0.87 |
| 10 | Cnbp | NM_013493 | 0.095 | 0.88 | 1.14 |
| 11 | Rab1 | NM_008996 | 0.074 | 0.86 | 1.17 |
| 12 | Atp6v1c1 | NM_025494 | 0.055 | 0.83 | 1.20 |
| 13 | Fnta | NM_008033 | 0.055 | 0.82 | 1.22 |
| 14 | Eno3 | ENSMUST00000108548 | 0.095 | 0.79 | 1.27 |
| 15 | Lass4 | NM_026058 | 0.077 | 0.72 | 1.39 |
| 16 | Pfkp | ENSMUST00000138703 | 0.095 | 0.69 | 1.45 |
| 17 | Snord73a | NR_004417 | 0.074 | 0.66 | 1.51 |
| 18 | Hydin | NM_172916 | 0.001 | 0.63 | 1.60 |

B. *Klf3*^H275R^ homozygotes (n=3) versus CH homozygotes (n=4)

|  | **Gene Symbol** | **RefSeq** | **FDR** | **H275R Homo**  **CH Homo** | **CH Homo**  **H275R Homo** |
| --- | --- | --- | --- | --- | --- |
| 1 | Klf3 | NM_008453 | 0.000 | 13.62 | 0.07 |
| 2 | Myh9 | NM_022410 | 0.032 | 1.61 | 0.62 |
| 3 | Magee2 | NM_053206 | 0.068 | 1.47 | 0.68 |
| 4 | Bcam | NM_020486 | 0.025 | 1.38 | 0.73 |
| 5 | Zfp579 | ENSMUST00000162731 | 0.014 | 1.35 | 0.74 |
| 6 | F2rl2 | ENSMUST00000022182 | 0.006 | 1.29 | 0.77 |
| 7 | Pias3 | NM_146135 | 0.018 | 1.25 | 0.80 |
| 8 | Zfp382 | NM_001081007 | 0.063 | 1.23 | 0.81 |
| 9 | Gm5885 | NM_001185040 | 0.082 | 1.23 | 0.82 |
| 10 | Atp6v1c1 | NM_025494 | 0.072 | 0.83 | 1.21 |
| 11 | Hydin | NM_172916 | 0.003 | 0.66 | 1.52 |
| 12 | Snord73a | NR_004417 | 0.068 | 0.63 | 1.59 |
| 13 | Coq2 | NM_027978 | 0.032 | 0.59 | 1.69 |
| 14 | Gimap4 | NM_174990 | 0.003 | 0.48 | 2.07 |
| 15 | Snora31 | NR_028481 | 0.072 | 0.46 | 2.20 |
| 16 | Fah | NM_010176 | 0.018 | 0.43 | 2.31 |

C. *Klf3*^H275R^ heterozygotes (n=4) versus CH homozygotes (n=4)

|  | **Gene Symbol** | **RefSeq** | **FDR** | **H275R Het**  **CH Homo** | **CH Homo**  **H275R Het** |
| --- | --- | --- | --- | --- | --- |
| 1 | Klf3 | NM_008453 | 0.000 | 15.02 | 0.07 |
| 2 | Hsd3b6 | NM_013821 | 0.036 | 2.65 | 0.38 |
| 3 | Mir1948 | NR_035471 | 0.084 | 2.08 | 0.48 |
| 4 | Lyrm7 | ENSMUST00000020506 | 0.096 | 1.87 | 0.53 |
| 5 | Mxra7 | ENSMUST00000021170 | 0.038 | 1.81 | 0.55 |
| 6 | Myh9 | NM_022410 | 0.012 | 1.67 | 0.60 |
| 7 | Gm10524 | AK147472 | 0.075 | 1.64 | 0.61 |
| 8 | Tubg2 | NM_134028 | 0.042 | 1.58 | 0.63 |
| 9 | Cyb5r3 | ENSMUST00000018186 | 0.032 | 1.55 | 0.65 |
| 10 | Nat6 | ENSMUST00000093785 | 0.060 | 1.53 | 0.66 |
| 11 | Slc43a2 | NM_001199284 | 0.094 | 1.50 | 0.67 |
| 12 | Grasp | NM_019518 | 0.083 | 1.49 | 0.67 |
| 13 | Magee2 | NM_053206 | 0.029 | 1.48 | 0.68 |
| 14 | Tubb4a | NM_009451 | 0.073 | 1.48 | 0.68 |
| 15 | D630041G03Rik | NR_028416 | 0.084 | 1.48 | 0.68 |
| 16 | Pkmyt1 | NM_023058 | 0.066 | 1.45 | 0.69 |
| 17 | Ncan | NM_007789 | 0.032 | 1.45 | 0.69 |
| 18 | 2310045N01Rik | ENSMUST00000002418 | 0.066 | 1.43 | 0.70 |
| 19 | Fam71e1 | NM_028169 | 0.075 | 1.42 | 0.71 |
| 20 | Qpctl | NM_026111 | 0.075 | 1.41 | 0.71 |
| 21 | Mrgpre | NM_175534 | 0.094 | 1.41 | 0.71 |
| 22 | Oscar | NM_175632 | 0.094 | 1.41 | 0.71 |
| 23 | Mark4 | NM_172279 | 0.094 | 1.40 | 0.71 |
| 24 | Zic3 | NM_009575 | 0.066 | 1.39 | 0.72 |
| 25 | Zfp235 | NM_019941 | 0.095 | 1.38 | 0.72 |
| 26 | A830005F24Rik | AK043525 | 0.090 | 1.37 | 0.73 |
| 27 | Zfhx2 | NM_001039198 | 0.096 | 1.36 | 0.74 |
| 28 | Frmd5 | NM_172673 | 0.073 | 1.36 | 0.74 |
| 29 | Csf2ra | NM_009970 | 0.082 | 1.36 | 0.74 |
| 30 | Man1c1 | NM_207237 | 0.060 | 1.35 | 0.74 |
| 31 | Ehd3 | NM_020578 | 0.080 | 1.35 | 0.74 |
| 32 | 6330415G19Rik | AK031835 | 0.082 | 1.35 | 0.74 |
| 33 | Gadd45gip1 | ENSMUST00000036734 | 0.075 | 1.35 | 0.74 |
| 34 | Bmyc | NM_023326 | 0.060 | 1.34 | 0.75 |
| 35 | Ncan | NM_007789 | 0.038 | 1.34 | 0.75 |
| 36 | Cnpy3 | NM_028065 | 0.090 | 1.33 | 0.75 |
| 37 | Lbx2 | NM_010692 | 0.086 | 1.32 | 0.75 |
| 38 | Prss27 | NM_175440 | 0.083 | 1.32 | 0.76 |
| 39 | Ccdc106 | NM_146178 | 0.049 | 1.31 | 0.76 |
| 40 | Bcl2 | NM_009741 | 0.071 | 1.31 | 0.76 |
| 41 | C130021I20Rik | NR_046275 | 0.090 | 1.29 | 0.77 |
| 42 | Cdh3 | NM_001037809 | 0.083 | 1.29 | 0.77 |
| 43 | Gatc | NM_029645 | 0.068 | 1.29 | 0.78 |
| 44 | C030046I01Rik | NM_177994 | 0.095 | 1.29 | 0.78 |
| 45 | Fmod | ENSMUST00000048183 | 0.095 | 1.28 | 0.78 |
| 46 | Cd276 | NM_133983 | 0.094 | 1.28 | 0.78 |
| 47 | Shc3 | NM_009167 | 0.060 | 1.28 | 0.78 |
| 48 | Slc9a3r2 | NM_023055 | 0.031 | 1.27 | 0.79 |
| 49 | 2210016L21Rik | BC031162 | 0.092 | 1.26 | 0.79 |
| 50 | Syde1 | NM_027875 | 0.018 | 1.26 | 0.79 |
| 51 | Nr4a2 | ENSMUST00000028166 | 0.082 | 1.26 | 0.79 |
| 52 | Zdhhc18 | ENSMUST00000084238 | 0.080 | 1.26 | 0.79 |
| 53 | Gm19304 | AK142452 | 0.086 | 1.25 | 0.80 |
| 54 | D7Ertd443e | NM_001199941 | 0.083 | 1.24 | 0.81 |
| 55 | Cyb5b | NM_025558 | 0.054 | 1.24 | 0.81 |
| 56 | Lrfn1 | NM_001141921 | 0.090 | 1.24 | 0.81 |
| 57 | Trim45 | NM_001165953 | 0.060 | 1.22 | 0.82 |
| 58 | Atp5g1 | ENSMUST00000090541 | 0.060 | 1.22 | 0.82 |
| 59 | Vmn2r85 | NM_001102602 | 0.095 | 1.22 | 0.82 |
| 60 | Nefh | NM_010904 | 0.066 | 1.21 | 0.82 |
| 61 | Phyhipl | NM_178621 | 0.039 | 1.21 | 0.83 |
| 62 | S1pr3 | NM_010101 | 0.090 | 1.21 | 0.83 |
| 63 | Bcam | NM_020486 | 0.096 | 1.20 | 0.83 |
| 64 | Fbxw5 | NM_013908 | 0.084 | 1.20 | 0.83 |
| 65 | Cenpv | NM_028448 | 0.095 | 1.20 | 0.83 |
| 66 | Mrvi1 | ENSMUST00000005751 | 0.060 | 1.20 | 0.83 |
| 67 | Btbd11 | NM_028709 | 0.038 | 1.20 | 0.83 |
| 68 | Lrrc4b | NM_198250 | 0.057 | 1.20 | 0.84 |
| 69 | Chst2 | NM_018763 | 0.020 | 1.20 | 0.84 |
| 70 | Vamp2 | NM_009497 | 0.086 | 1.20 | 0.84 |
| 71 | 9530051G07Rik | NR_040272 | 0.077 | 1.19 | 0.84 |
| 72 | Zfp579 | ENSMUST00000162731 | 0.082 | 1.19 | 0.84 |
| 73 | Lrrc57 | NM_001159612 | 0.080 | 1.19 | 0.84 |
| 74 | Opalin | ENSMUST00000087176 | 0.075 | 1.18 | 0.84 |
| 75 | F2rl2 | ENSMUST00000022182 | 0.036 | 1.18 | 0.85 |
| 76 | Lix1l | NM_001163170 | 0.095 | 1.18 | 0.85 |
| 77 | Robo1 | NM_019413 | 0.032 | 1.17 | 0.85 |
| 78 | Btrc | NM_001037758 | 0.032 | 1.17 | 0.86 |
| 79 | Pias3 | NM_146135 | 0.055 | 1.16 | 0.86 |
| 80 | Limch1 | NM_001001980 | 0.060 | 1.16 | 0.86 |
| 81 | Zfp382 | NM_001081007 | 0.077 | 1.16 | 0.86 |
| 82 | Ptchd2 | NM_001083342 | 0.083 | 1.15 | 0.87 |
| 83 | Dpysl3 | AF501324 | 0.095 | 1.15 | 0.87 |
| 84 | Dguok | NM_013764 | 0.031 | 1.15 | 0.87 |
| 85 | 5730494M16Rik | NM_001004361 | 0.031 | 1.15 | 0.87 |
| 86 | Rnpep | NM_145417 | 0.077 | 1.15 | 0.87 |
| 87 | Rbfox3 | NM_001039167 | 0.061 | 1.14 | 0.87 |
| 88 | Amot | ENSMUST00000112836 | 0.062 | 1.13 | 0.88 |
| 89 | Atp5d | ENSMUST00000105367 | 0.042 | 1.11 | 0.90 |
| 90 | Timm23 | NM_016897 | 0.094 | 0.88 | 1.13 |
| 91 | Dync1li2 | NM_001013380 | 0.092 | 0.88 | 1.14 |
| 92 | Polr3a | NM_001081247 | 0.090 | 0.88 | 1.14 |
| 93 | Slc30a9 | ENSMUST00000162372 | 0.034 | 0.87 | 1.15 |
| 94 | Anapc4 | ENSMUST00000031072 | 0.088 | 0.86 | 1.16 |
| 95 | Stmn1 | ENSMUST00000105868 | 0.075 | 0.86 | 1.16 |
| 96 | Usp8 | NM_001252580 | 0.095 | 0.86 | 1.17 |
| 97 | Cnbp | NM_013493 | 0.031 | 0.86 | 1.17 |
| 98 | Inpp5k | ENSMUST00000006286 | 0.082 | 0.86 | 1.17 |
| 99 | Sdad1 | NM_172713 | 0.082 | 0.85 | 1.17 |
| 100 | Rab1 | NM_008996 | 0.033 | 0.85 | 1.17 |
| 101 | Rps12 | NM_011295 | 0.062 | 0.85 | 1.18 |
| 102 | Tm9sf3 | NM_133352 | 0.036 | 0.85 | 1.18 |
| 103 | Acp1 | NM_001110239 | 0.090 | 0.85 | 1.18 |
| 104 | Cdc37l1 | NM_025950 | 0.095 | 0.85 | 1.18 |
| 105 | As3mt | NM_020577 | 0.087 | 0.84 | 1.18 |
| 106 | Mtx2 | ENSMUST00000028511 | 0.032 | 0.84 | 1.19 |
| 107 | Arl1 | ENSMUST00000116234 | 0.094 | 0.84 | 1.19 |
| 108 | Plekha1 | NM_133942 | 0.080 | 0.84 | 1.19 |
| 109 | Ubr1 | NM_009461 | 0.036 | 0.84 | 1.19 |
| 110 | Atp6v1c1 | NM_025494 | 0.036 | 0.84 | 1.19 |
| 111 | Ndfip2 | NM_029561 | 0.082 | 0.84 | 1.19 |
| 112 | Vps4b | ENSMUST00000112736 | 0.075 | 0.84 | 1.19 |
| 113 | Ndufa5 | ENSMUST00000023851 | 0.060 | 0.83 | 1.20 |
| 114 | Unc50 | NM_026123 | 0.096 | 0.83 | 1.20 |
| 115 | Tspan12 | NM_173007 | 0.032 | 0.83 | 1.20 |
| 116 | Ociad1 | NM_023429 | 0.017 | 0.83 | 1.21 |
| 117 | Sft2d1 | NM_134114 | 0.031 | 0.83 | 1.21 |
| 118 | Tpm3 | NM_022314 | 0.095 | 0.83 | 1.21 |
| 119 | Zbtb44 | NM_001115130 | 0.066 | 0.82 | 1.21 |
| 120 | Fnta | NM_008033 | 0.034 | 0.82 | 1.22 |
| 121 | Uchl5 | NM_019562 | 0.031 | 0.82 | 1.22 |
| 122 | Mdm4 | NM_008575 | 0.060 | 0.82 | 1.22 |
| 123 | Gnl3 | NM_153547 | 0.075 | 0.81 | 1.23 |
| 124 | Tmx1 | ENSMUST00000021471 | 0.066 | 0.81 | 1.24 |
| 125 | Ccnl2 | NM_207678 | 0.075 | 0.81 | 1.24 |
| 126 | Galnt11 | ENSMUST00000114952 | 0.096 | 0.80 | 1.24 |
| 127 | Klhl2 | NM_178633 | 0.066 | 0.80 | 1.24 |
| 128 | Snora17 | NR_028571 | 0.034 | 0.80 | 1.25 |
| 129 | Stard4 | ENSMUST00000025236 | 0.095 | 0.80 | 1.25 |
| 130 | Tprkb | NM_001170488 | 0.098 | 0.80 | 1.25 |
| 131 | Emr4 | NM_139138 | 0.082 | 0.80 | 1.25 |
| 132 | 1700031L13Rik | AK006583 | 0.095 | 0.80 | 1.25 |
| 133 | Pacrgl | NM_025755 | 0.082 | 0.80 | 1.25 |
| 134 | 2610005L07Rik | NR_028428 | 0.083 | 0.80 | 1.25 |
| 135 | Car13 | NM_024495 | 0.052 | 0.80 | 1.26 |
| 136 | Med26 | AK054023 | 0.095 | 0.80 | 1.26 |
| 137 | Rpl9 | NM_011292 | 0.060 | 0.79 | 1.26 |
| 138 | Luc7l3 | NM_026313 | 0.082 | 0.79 | 1.27 |
| 139 | 9430037G07Rik | NR_040766 | 0.094 | 0.79 | 1.27 |
| 140 | Srsf5 | NM_001079695 | 0.064 | 0.78 | 1.27 |
| 141 | Trnt1 | NM_001242358 | 0.054 | 0.78 | 1.28 |
| 142 | Atp2c1 | NM_175025 | 0.031 | 0.78 | 1.28 |
| 143 | Dsc2 | ENSMUST00000039247 | 0.066 | 0.78 | 1.28 |
| 144 | Rpl12 | BC075731 | 0.032 | 0.77 | 1.30 |
| 145 | Pcna | ENSMUST00000028817 | 0.055 | 0.77 | 1.30 |
| 146 | Tmem87a | NM_173734 | 0.087 | 0.77 | 1.30 |
| 147 | Snhg4 | NR_038073 | 0.062 | 0.77 | 1.31 |
| 148 | Nop58 | ENSMUST00000027174 | 0.094 | 0.77 | 1.31 |
| 149 | Zdhhc2 | ENSMUST00000049389 | 0.094 | 0.76 | 1.31 |
| 150 | Sumf1 | NM_145937 | 0.075 | 0.76 | 1.31 |
| 151 | Myf6 | NM_008657 | 0.060 | 0.76 | 1.32 |
| 152 | 1700034H14Rik | NM_025969 | 0.071 | 0.75 | 1.33 |
| 153 | Eno3 | ENSMUST00000108548 | 0.029 | 0.75 | 1.33 |
| 154 | Parp4 | NM_001145978 | 0.051 | 0.74 | 1.35 |
| 155 | Wdfy1 | NM_001111279 | 0.041 | 0.74 | 1.35 |
| 156 | Pon3 | NM_173006 | 0.066 | 0.74 | 1.35 |
| 157 | Snhg1 | NR_002896 | 0.066 | 0.73 | 1.36 |
| 158 | Mysm1 | NM_177239 | 0.095 | 0.73 | 1.37 |
| 159 | 1190002H23Rik | NM_025427 | 0.094 | 0.73 | 1.38 |
| 160 | Ccdc36 | NM_001135198 | 0.083 | 0.73 | 1.38 |
| 161 | Lass4 | NM_026058 | 0.041 | 0.73 | 1.38 |
| 162 | 2900076A07Rik | NR_045299 | 0.094 | 0.72 | 1.38 |
| 163 | Zfp568 | NM_001167872 | 0.042 | 0.72 | 1.39 |
| 164 | Pfkp | ENSMUST00000138703 | 0.075 | 0.72 | 1.39 |
| 165 | AU019823 | NM_001134902 | 0.036 | 0.72 | 1.39 |
| 166 | 1810032O08Rik | NR_027821 | 0.032 | 0.71 | 1.41 |
| 167 | Tmem20 | NM_175507 | 0.083 | 0.71 | 1.41 |
| 168 | Gm20255 | AK133679 | 0.090 | 0.70 | 1.42 |
| 169 | Hydin | NM_172916 | 0.003 | 0.69 | 1.46 |
| 170 | Snord22 | NR_004445 | 0.083 | 0.68 | 1.46 |
| 171 | Sfxn2 | NM_053196 | 0.041 | 0.67 | 1.48 |
| 172 | Psma8 | NM_001163609 | 0.090 | 0.66 | 1.51 |
| 173 | Zfp868 | NM_001045553 | 0.084 | 0.66 | 1.52 |
| 174 | Coq2 | NM_027978 | 0.036 | 0.65 | 1.54 |
| 175 | Snhg3 | NR_003270 | 0.080 | 0.64 | 1.56 |
| 176 | 2610005L07Rik | NR_028428 | 0.083 | 0.61 | 1.64 |
| 177 | Snord73a | NR_004417 | 0.020 | 0.60 | 1.65 |
| 178 | Gimap4 | NM_174990 | 0.018 | 0.60 | 1.68 |
| 179 | Fah | NM_010176 | 0.061 | 0.58 | 1.73 |
| 180 | Snora75 | NR_028478 | 0.051 | 0.58 | 1.74 |
| 181 | Rps3a | BC084675 | 0.061 | 0.52 | 1.92 |
| 182 | Mir690 | NR_030463 | 0.091 | 0.52 | 1.93 |
| 183 | Crip1 | NM_007763 | 0.073 | 0.51 | 1.98 |
| 184 | Snora31 | NR_028481 | 0.038 | 0.49 | 2.04 |
| 185 | Slc6a16 | XM_914689 | 0.083 | 0.45 | 2.24 |
