# Supplementary material for: ENU-induced Mutation in the DNA-binding Domain of KLF3 Reveals Important Roles for KLF3 in Cardiovascular Development and Function in Mice
Source: PLoS Genet. 2013 Jul 11;9(7):e1003612. doi: 10.1371/journal.pgen.1003612 (PMC3708807; doi:10.1371/journal.pgen.1003612)
Supplement: Table S7 — PCR primers for genomic DNA used to genotype mouse lines. (DOCX) [file pgen.1003612.s019.docx]

**Table S7**. PCR primers for genomic DNA used to genotype mouse lines.

| **Mutant** | **Allele** | **Forward** | **Reverse** | **Product (bp)** |
| --- | --- | --- | --- | --- |
| *Klf3* | WT | 5’-TTGAGGGAAGGGAAGCACCTTT-3’ | 5’-TAAGAACGACCCACCCAACTCA-3’ | 301* |
|  | H275R | Same as WT *Klf3* | Same as WT *Klf3* | 187*  114* |
| XS0187 | WT | 5’-ATGAGGTCTTGGGATGGTCA-3’ | 5’-AGAGACTGAGCCCGTCTGACC-3’ | 604 |
|  | Insertion | 5’-ATGAGGTCTTGGGATGGTCA-3’ | 5’-CTACTTGTCTGGAGGCCAGG-3’ | 292 |
| CH0516 | WT | 5’-GGCCTCCACAGTCTGGATAA-3’ | 5’-CACAAGGCATGGGAACAA-3’ | 764 |
|  | Insertion | 5’-GGCCTCCACAGTCTGGATAA-3’ | 5’-CATAGGAGCCAGGTCCCTCT-3’ | 409 |

*301 bp product was treated with BstUII (37^o^C for 8h); WT DNA fragment remains 301 bp in size whereas mutant DNA is digested into 2 fragments of 187 bp and 114 bp in size.
