# Supplementary material for: ENU-induced Mutation in the DNA-binding Domain of KLF3 Reveals Important Roles for KLF3 in Cardiovascular Development and Function in Mice
Source: PLoS Genet. 2013 Jul 11;9(7):e1003612. doi: 10.1371/journal.pgen.1003612 (PMC3708807; doi:10.1371/journal.pgen.1003612)
Supplement: Table S8 — qRT-PCR primer sequences for mRNA. (DOCX) [file pgen.1003612.s020.docx]

**Table S8.** qRT-PCR primer sequences for mRNA.

| **Gene** | **Forward** | **Reverse** |
| --- | --- | --- |
| *Klf3* | 5̕’-GAAATGTCACCCCCTTTAATGAAC-3̕’ | 5̕’-CACGATGACGGAAGGATGGT-3̕’ |
| *Lgals3* | 5̕’-GTCCCCGCTGGACCACTGAC-3̕’ | 5̕’-CCTCCAGGCAAGGGCAGGTC-3̕’ |
| *Hsd3b6* | 5̕’-CAGTGTTCCAGCCTTCATCTTCT-3̕’ | 5̕’-TGCCATTCAGGATTATCTCCTTGT-3̕’ |
| *Lilra6* | 5̕’-CAGAGCTATCACAGGTTCATTTTAACTG-3̕’ | 5̕’-GGCGTGGTACTTCCCTGTAGAG-3̕’ |
| *18S* | 5̕’-CACGGCCGGTACAGTGAAAC-3̕’ | 5̕’-AGAGGAGCGAGCGACCAA-3̕’ |
